# Chemical Game Theory: Linking Metabolite Diversity, Biosynthetic Architecture, and Realized Payoffs

**DOI:** 10.3390/molecules31183325

**Published:** 2026-09-19

**Authors:** Davyson de Lima Moreira, Daniel de Brito Machado, Ygor Jessé Ramos, Renato Crespo Pereira

**Affiliations:** 1 Research Institute of the Rio de Janeiro Botanical Garden, Rua Pacheco Leão 915, Jardim Botânico, Rio de Janeiro 22460-030, RJ, Brazil; danielmachado.cedae@gmail.com; 2 Farmácia da Terra Laboratory, Faculty of Pharmacy, Federal University of Bahia, Rua Barão de Jeremoabo, 147, Ondina, Salvador 40170-115, BA, Brazil; ygor.jesse@ufba.br; 3 Department of Marine Biology, Universidade Federal Fluminense, Campus do Valonguinho, Outeiro de São João Batista, s/n, Centro, Niterói 24020-141, RJ, Brazil; rcrespo@id.uff.br

**Keywords:** bioprospection, phytochemical prediction, chemodiversity, ontogeny, Piperaceae, *Piper mollicomum*

## Abstract

Plant chemodiversity is generally evaluated through metabolite richness, relative abundance, compositional dissimilarity, and biosynthetic organization. However, these descriptors characterize chemical states without explicitly identifying which metabolites, chemical classes, or biosynthetic pathways gain or lose relative representation during transitions between states. Here, we introduce Chemical Game Theory as an operational analogy inspired by the relative-performance principle of replicator dynamics. Metabolites are treated as elementary chemical strategies, biosynthetic pathways constitute higher-order strategies, and normalized chromatographic abundances define their frequencies within a mixture. A centered log-ratio quantity, termed realized chemical payoff within this framework, retrospectively quantifies whether a component gained or lost proportional representation relative to the abundance-weighted mean log change in the system. It is not inferred from a payoff matrix and does not represent Darwinian fitness, interaction strength, or absolute biosynthetic production. Shannon diversity describes metabolite-level coexistence, whereas the General Biosynthetic Diversity Index, GBDI, characterizes abundance-weighted pathway organization and intrapathway diversification. The framework was applied to previously published GC-MS and GC-FID profiles of essential oils from leaves and four developmental stages of the reproductive organ of *Piper mollicomum* Kunth, sampled over five months. Leaves had the highest numerical mean Shannon diversity and GBDI, although the Shannon difference was not statistically significant. The reproductive stages followed temporally variable compositional trajectories. Across the five descriptive monthly blocks, the terpenoid route gained relative representation during the Stage I to II and Stage III to IV transitions, whereas the mixed category declined. Chemical dominance, Shannon diversity, GBDI, and realized payoff captured related but non-equivalent dimensions of relative chemical organization. As a proof of concept, Chemical Game Theory provides a quantitative language for retrospective analysis of compositional redistribution. Its proposed use for prioritizing plant material and its transferability to other chromatographic platforms require validation with independent datasets, biological replication, absolute quantification, and bioactivity-guided experiments.

## 1. Introduction

Plants produce chemically complex and dynamic mixtures of specialized metabolites whose composition varies among species, populations, individuals, organs, developmental stages, and environmental conditions. These metabolites participate in defense, signaling, reproduction, stress tolerance, and interactions with other organisms, while also representing a major source of structurally and biologically diverse natural products [1,2,3,4,5,6]. Plant chemodiversity should therefore not be regarded as a static inventory of molecular structures, but as a dynamic chemical phenotype generated by the differential allocation of metabolic resources among precursors, enzymes, biosynthetic pathways, and their final products [4,5,6].

Quantitative studies of chemodiversity commonly employ metabolite richness, Shannon and Simpson indices, Hill numbers, evenness, chemical dissimilarities, and metrics incorporating structural, functional, or biosynthetic information [4,5,6,7,8]. These approaches provide valuable information about the number of metabolites, their abundance distribution, and the degree of differentiation among chemical mixtures [5,6,7,8]. However, classical diversity indices generally treat metabolites as independent and exchangeable units, thereby overlooking the nested metabolic relationships that connect chemical products through common precursor pools, catalytic machinery, regulatory controls, and biosynthetic costs [7,8]. Accordingly, comparable Shannon values can emerge from mixtures characterized by fundamentally different patterns of pathway allocation and internal metabolic branching [8].

The General Biosynthetic Diversity Index, GBDI, was developed to address this distinction by coupling the abundance of each metabolite with the cumulative abundance of its assigned biosynthetic pathway [8]. GBDI integrates allocation among biosynthetic pathways with diversification occurring within each pathway, thereby distinguishing compound-level diversity from biosynthetic architectural diversity. A mixture dominated by one pathway may still exhibit a relatively high GBDI when that pathway is internally branched into several quantitatively relevant products. Conversely, a mixture containing several pathways may remain architecturally canalized if most chemical abundance is concentrated in a single compound or route. Shannon diversity and GBDI therefore describe complementary properties of chemical organization.

Although these descriptors quantify the structure of a chemical state, they do not directly indicate which chemical components gain or lose relative representation during transitions between states [5,6,7,8]. This distinction is important because chemical dominance and directional advantage are not equivalent. A highly abundant metabolite may remain dominant while already decreasing proportionally, whereas a minor compound or pathway may exhibit rapid relative expansion before becoming quantitatively prominent. Static comparisons can identify compositional differences, but they do not formally quantify the direction and intensity of compositional redistribution.

Game theory provides a mathematical framework for systems in which the performance of a strategy depends on the composition and behavior of the surrounding system [9,10,11,12,13,14,15,16]. In evolutionary game theory, strategies do not need to correspond to conscious decisions. They may represent phenotypes, resource-allocation patterns, functional traits, or other alternatives whose relative frequencies change according to their performance [11,12,13,15,16]. Replicator dynamics formalize this principle by predicting that strategies with performance above the system-wide mean increase in frequency, whereas strategies with below-average performance decline [11,12,13,15,16].

However, classical non-cooperative game theory defines a Nash equilibrium as a strategy profile in which no player can improve its payoff through unilateral deviation while the strategies of the other players remain unchanged [14]. The present framework does not estimate a Nash equilibrium, infer a payoff matrix, or recover interaction coefficients among metabolites. Instead, it adopts the relative-performance principle of replicator dynamics as an operational analogy [15,16]. 

For the continuous replicator equation,

$$\frac{d\ln p_{i}}{dt}=f_{i}-\bar{f},$$

where $f_{i}$ is the fitness of type i and $\bar{f}$ is the population mean fitness. Integrating this expression over an interval gives

$$\ln\left[\frac{p_{i}\left(t+1\right)}{p_{i}\left(t\right)}\right]$$

as the accumulated fitness difference when the replicator assumptions hold. In the present retrospective chemical translation, the observed discrete log-ratio

$$r_{i}\left(t\right)=\ln\left[\frac{p_{i}\left(t+1\right)}{p_{i}\left(t\right)}\right]$$

is measured directly and then centered by the initial-abundance-weighted mean log change:

$$g_{i}\left(t\right)=r_{i}\left(t\right)-\sum_{j}p_{j}\left(t\right)r_{j}\left(t\right).$$

Therefore, $g_{i}$ is a centered relative change defined as a realized chemical payoff within the proposed framework, not as an independently estimated conventional game payoff or Darwinian fitness. It quantifies relative compositional enrichment or depletion between measured states and does not identify the biological process that produced the change.

Game-theoretical models have been applied to plant competition, resource acquisition, defense allocation, herbivory tolerance, allelopathy, and chemical communication [17,18,19,20,21]. Game theory has also contributed to theoretical discussions concerning the evolution and maintenance of plant chemodiversity, particularly through frequency-dependent interactions among defended and undefended plants or among plants, herbivores, and competing organisms [22,23,24,25,26]. In most of these models, however, the strategies correspond to organisms, phenotypes, or defense-investment levels. Chromatographically measured metabolites and biosynthetic pathways have rarely been operationalized as frequency-distributed strategies whose directional performance can be calculated directly from empirical chemical compositions.

**Figure 1:**
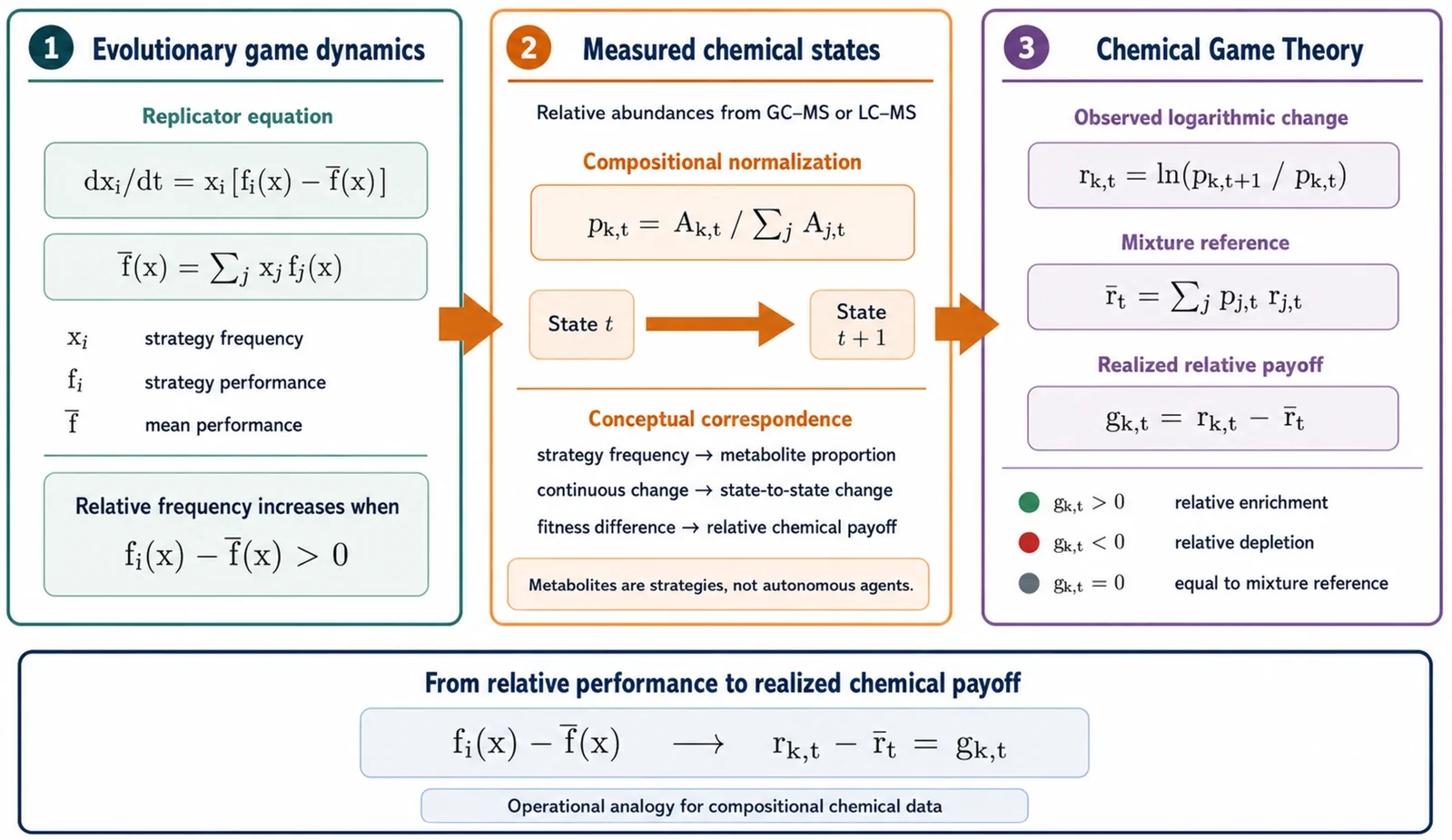
Conceptual translation from evolutionary game dynamics to Chemical Game Theory. The revised figure preserves the original three-step conceptual correspondence while increasing the size and legibility of all labels and equations. The replicator equation describes changes in strategy frequency according to the difference between individual and mean population performance. In Chemical Game Theory, normalized chromatographic abundance represents the proportional participation of a metabolite in a measured chemical state. Continuous evolutionary change is translated operationally into an observed logarithmic change between consecutive states, which is centered by the abundance-weighted mixture reference to obtain the realized relative payoff. Positive and negative values indicate proportional enrichment and depletion relative to that reference, respectively. Metabolites are treated as indexed chemical strategies within a compositional system, not as autonomous biological agents.

**Figure 2:**
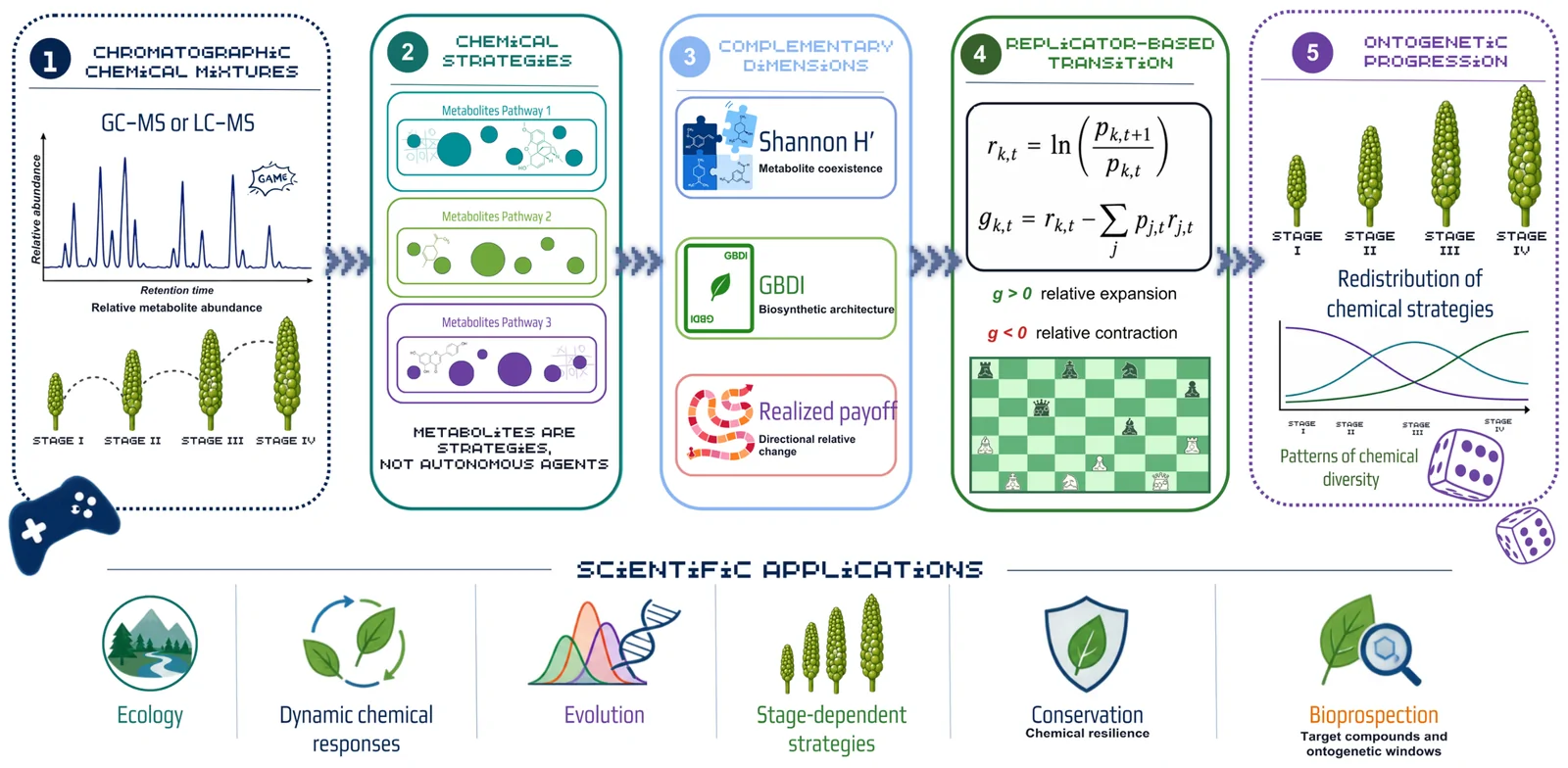
Conceptual framework of Chemical Game Theory applied to plant chemodiversity. Chromatographic constituents are represented as elementary chemical strategies, whereas biosynthetic pathways constitute higher-order strategies. Shannon diversity describes metabolite coexistence; the General Biosynthetic Diversity Index, GBDI, characterizes biosynthetic architecture; and realized payoffs quantify centered relative changes between consecutive chemical states. The scheme is conceptual and does not represent experimental observations, generated data, or inferential results. Its scientific content, labels, and correspondence with the mathematical definitions were verified by the authors.

Here, we introduce Chemical Game Theory (Figure 1 and Figure 2) as an operational framework for retrospective analysis of dynamic plant chemodiversity. A chemical component is termed a player only as an indexed unit in the compositional vector. A chemical strategy is a metabolite, chemical class, or biosynthetic category whose normalized proportion is followed across ordered states. Realized chemical payoff is the centered log-ratio change defined by the model. Chemical allocation denotes only the observed fraction of the closed chromatographic profile assigned to a component or category. Strategic emergence denotes positive relative change combined with low initial representation and is treated as a candidate analytical pattern rather than evidence of competition, selection, behavior, or increased absolute production.

These operational terms do not attribute agency to metabolites. Their relative abundances are observable outcomes that may be influenced by production, precursor availability, enzymatic regulation, transport, storage, volatilization, degradation, extraction, and detector response. Because the present data are relative, the framework cannot distinguish among these mechanisms. Accordingly, terms such as biosynthetic investment, active biosynthetic window, and biosynthetic reorganization have been replaced by relative representation, candidate relative-enrichment window, and compositional reorganization where absolute production was not measured.

The proposed framework separates four complementary dimensions of chemical organization. Chemical dominance identifies the most abundant compounds or pathways. Shannon diversity describes metabolite-level coexistence and abundance distribution. GBDI characterizes the abundance-weighted organization of metabolites within and among biosynthetic pathways. Realized chemical payoff quantifies the direction and intensity with which a strategy changes between consecutive chemical states. These dimensions may be related, but they are not interchangeable. A mixture may exhibit high GBDI and low Shannon diversity, or a rare pathway may present a positive payoff without becoming quantitatively dominant.

Developmental and organ-specific chemical variation provides a particularly suitable context for demonstrating the framework. Plant organs differ in physiological function, tissue value, resource availability, storage capacity, and exposure to biotic and abiotic pressures [24,25,26]. Reproductive development may involve changes in enzymatic activity, precursor allocation, oxidation, volatilization, and the relative importance of defensive or signaling compounds. Consequently, successive stages of an organ may occupy distinct regions of compositional and biosynthetic space.

From a phytochemical perspective, these differences are directly relevant to natural-product discovery. Bioprospecting strategies frequently prioritize taxonomic identity, traditional use, preliminary biological activity, or the abundance of known compounds [27,28,29,30]. However, the organ and developmental stage selected for extraction may strongly influence the probability of obtaining a target metabolite, detecting minor compounds, or accessing a broader biosynthetic space. Developmental variation should therefore not be treated only as analytical noise. It may provide a rational criterion for selecting plant material.

Chemical Game Theory introduces a hypothesis for bioprospecting that must be tested prospectively. A plant compartment may be selected because it shows the highest observed relative abundance of a target compound, termed here a candidate target-yield state. Alternatively, a transition may be flagged because a target compound or annotated category gains proportional representation before reaching dominance, termed a candidate strategic-emergence state. The first criterion directly reflects relative chromatographic abundance. The second is an exploratory prioritization rule and has not yet been shown to improve isolation yield, structural novelty, or biological activity.

The framework may generate candidate priorities when combined with untargeted chemical profiling, dereplication, molecular-network analysis, absolute quantification, and bioactivity screening. High GBDI does not predict biological activity or structural novelty, and positive payoff does not demonstrate increased production. These descriptors may be used to formulate testable sampling hypotheses, which require prospective validation against extraction yield, compound isolation, and biological activity.

As a proof of concept, we applied Chemical Game Theory to previously published essential-oil data from *Piper mollicomum* Kunth [31]. The dataset includes leaves and four developmental stages of the reproductive organ sampled monthly from September to January. Essential oils were characterized by GC-MS and quantified by GC-FID. This design provides two complementary levels of comparison. Leaves serve as a vegetative reference for evaluating compartment-specific chemodiversity, while stages I–IV constitute an ordered reproductive trajectory that can be evaluated independently within each monthly block.

The biosynthetic classification used in the present analysis followed the precursor origin of the detected volatile constituents. Terpenoids were assigned to the terpene pathway, benzyl benzoate was assigned to shikimate-derived benzenoid metabolism, and eupatoriochromene was treated as a mixed-origin chromene. The latter assignment is supported by experimental studies of *Piper* chromenes showing incorporation of isoprene units from MVA- and MEP-derived metabolism and enzymatic prenylation of a chromene acceptor with dimethylallyl diphosphate [31,32,33,34,35,36,37,38,39,40,41,42,43,44,45,46]. Because pathway annotation affects pathway-sensitive descriptors, a counterfactual analysis assigning eupatoriochromene to either the terpenoid or shikimate category is provided in Supplementary File S1.

Accordingly, the objectives of this study were: (i) to formulate an operational game-theoretical analogy for chromatographic chemical mixtures; (ii) to define a centered log-ratio measure termed realized chemical payoff; (iii) to integrate this measure with Shannon diversity and GBDI; (iv) to compare the relative chemical organization of leaves and reproductive stages of *P. mollicomum*; (v) to describe stage transitions independently across five monthly blocks; and (vi) to formulate testable applications for future phytochemical and bioprospecting studies.

## 2. Results and Discussion

### 2.1. Construction of the Chemical Game from Ontogenetic Essential-Oil Profiles

The essential-oil dataset of *Piper mollicomum* provided an appropriate empirical system for demonstrating the proposed Chemical Game Theory framework because it combined chemical composition, biosynthetic organization, organ differentiation, and an ordered reproductive-development sequence. The original dataset comprised essential oils obtained from leaves and four developmental stages of the reproductive organ, sampled monthly from September 2020 to January 2021 [31]. See Supplementary File S1.

Each chromatographic profile was treated as a finite chemical system composed of S detected metabolites. The relative chromatographic abundance of metabolite i, denoted $p_{i}$, was interpreted as its realized allocation within the mixture. This interpretation recognizes that normalized chromatographic profiles are compositional: the components are constrained by a constant sum and, consequently, cannot vary independently [32,33,34].

For each chemical state, the relative abundances were normalized according to (Figure S1):

$$p_{i}=\frac{x_{i}}{\sum_{j=1}^{S}x_{j}}$$

and therefore:

$$\sum_{i=1}^{S}p_{i}=1$$

where $x_{i}$ is the chromatographic abundance originally reported for metabolite i.

Calculations were performed using the complete individual profiles reported as “% in 1” in Supplementary Table S3 of the original study [31]. These profiles were selected because they constituted internally reconcilable compositional vectors. The arithmetic averages reported for selected major constituents in other Supplementary Tables were retained for descriptive comparisons but were not used as closed input vectors because they did not include every detected constituent.

The metabolites were analyzed at three nested strategic levels:

1. Individual metabolites;
2. Chemical classes;
3. Biosynthetic pathways.

This hierarchical organization recognizes that plant specialized metabolism can be evaluated both through the behavior of individual products and through the broader precursor-generating and enzymatic systems responsible for their formation [23,24,25,26].

The four developmental stages constituted the ordered chemical-game trajectory:

Stage I→Stage II→Stage III→Stage IV

Leaves were treated as a vegetative reference compartment and not as an ontogenetic Stage 0. A leaf-to-reproductive-organ transition would combine two distinct biological effects, organ identity and developmental progression. Consequently, realized payoffs were calculated only between adjacent reproductive stages within the same monthly block. Leaves were compared with the reproductive stages using diversity, dominance, and biosynthetic-architecture descriptors (Tables S1 and S2).

In classical game theory, payoff describes an outcome associated with a strategy under a defined interaction structure. The present analysis does not estimate such an interaction structure. Chemical Game Theory instead uses the relative-performance form of replicator dynamics as an operational analogy for centered compositional change. The resulting realized chemical payoff is a model-defined retrospective descriptor, not a conventional payoff inferred from a game matrix.

Accordingly, chemical player, chemical strategy, and realized chemical payoff are operational terms defined in this article. They identify indexed components, aggregation levels, and centered relative changes, respectively. They do not demonstrate molecular agency, competition, cooperation, selection, behavior, causal interaction, Darwinian fitness, or absolute biosynthetic production.

### 2.2. Vegetative and Reproductive Compartments Occupied Distinct Regions of Chemical Diversity Space

**Figure 3:**
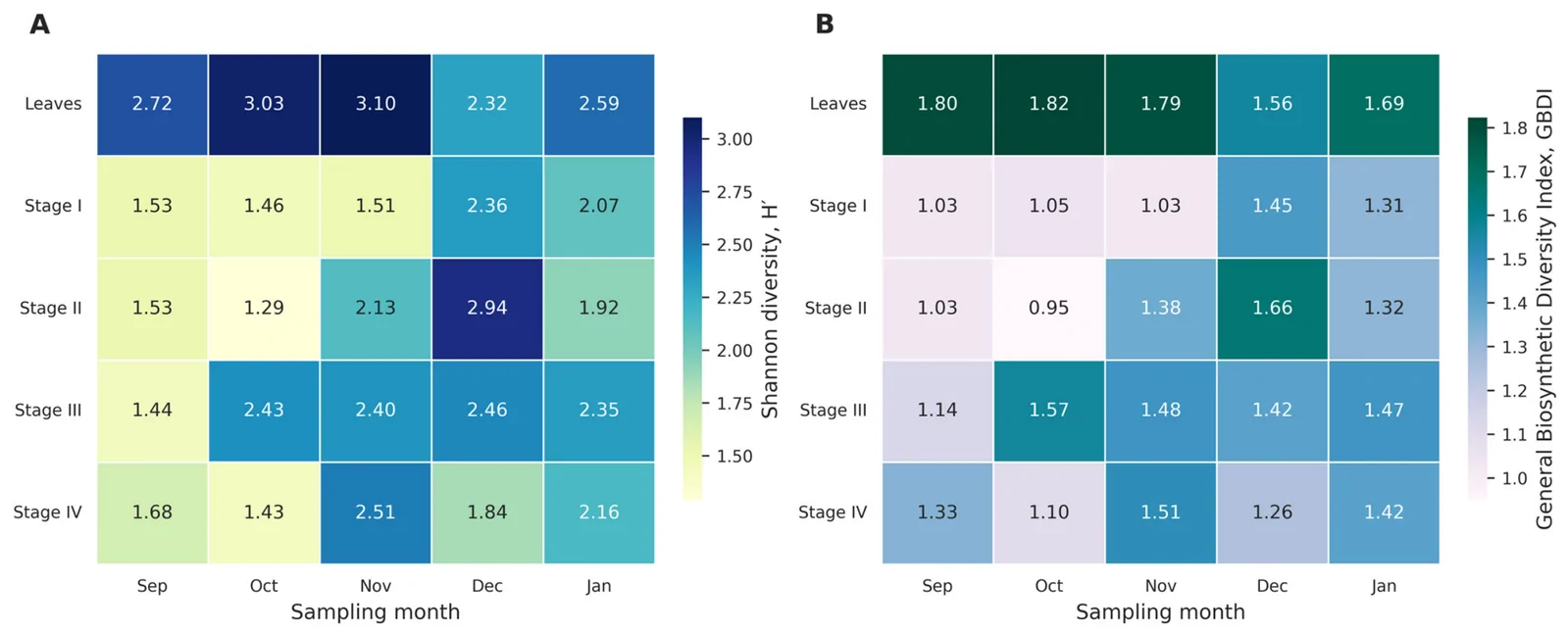
Compound-level Shannon diversity (**A**) and GBDI (**B**) across leaves and four reproductive stages of *Piper mollicomum*. Columns represent monthly sampling blocks. Higher Shannon values indicate greater metabolite richness and evenness, whereas GBDI additionally incorporates the distribution of metabolites within biosynthetic pathways [2,3,4,5,8]. Leaves were used as a vegetative reference and were not included in the ordered reproductive payoff trajectory.

Leaves showed the highest numerical mean Shannon diversity and GBDI among the evaluated compartments, H’ = 2.751 ± 0.322 and GBDI = 1.735 ± 0.108, respectively (Table 1 and Table S1; Figure 3). The qualifier numerical is essential because the omnibus Friedman test for Shannon diversity was not significant (*p* = 0.1933). Thus, the Shannon pattern is descriptive and does not establish a statistically supported difference among compartments.

Reproductive Stage I presented the lowest numerical mean Shannon diversity and GBDI, 1.787 ± 0.408 and 1.175 ± 0.195, respectively. Stage II and Stage III showed progressively larger numerical means, with Stage III presenting the highest numerical means among reproductive stages, H’ = 2.214 ± 0.435 and GBDI = 1.414 ± 0.163. These stage-specific contrasts are descriptive because no Holm-adjusted pairwise comparison was significant.

The transition from Stage III to Stage IV was accompanied by reductions in Shannon diversity and GBDI. Mean Shannon diversity declined from 2.214 to 1.925, and mean GBDI declined from 1.414 to 1.324. Simultaneously, the mean abundance of the dominant metabolite increased from 0.356 to 0.407. Together, these results indicate that late reproductive development was associated, on average, with greater concentration of chemical abundance around a smaller subset of quantitatively dominant metabolites.

The blocked Friedman analysis detected differences among compartments for GBDI ($\chi^{2}=10.72$, df=4, p=0.0299), pathway entropy ($\chi^{2}=17.12$, p=0.0018), and dominant-pathway abundance ($\chi^{2}=17.12$, p=0.0018). Shannon diversity did not differ significantly in the omnibus analysis (p=0.1933), despite its higher mean in leaves.

No pairwise Wilcoxon comparison remained significant after Holm correction. Consequently, no specific pair of compartments can be claimed to differ statistically in the present dataset. The significant omnibus tests for GBDI, pathway entropy, and dominant-pathway abundance indicate an overall rank difference across compartments, but the compartment-level patterns discussed below are descriptive. With five temporal blocks, all inferential results are exploratory and require independent replication.

### 2.3. Shannon Diversity, Pathway Entropy, and GBDI Represented Different Properties of the Chemical System

Although Shannon diversity and GBDI were strongly correlated across the 25 chemical states (Pearson r = 0.960, *p* < 0.001), they are mathematically non-equivalent rather than statistically independent. GBDI was moderately associated with pathway entropy (r = −0.537, *p* = 0.006) and dominant-pathway abundance (r = 0.533, *p* = 0.006). Shannon is calculated solely from compound proportions, whereas GBDI additionally depends on the pathway total assigned to each compound. These results support complementary interpretation but do not justify treating the descriptors as independent variables.

This distinction was particularly evident in leaves. Leaves exhibited the highest mean Shannon diversity and GBDI, although approximately 97.7% of their normalized chemical abundance belonged to the terpenoid pathway. Consequently, leaves displayed very low pathway entropy, $H_{path}=0.125$, and high dominant-pathway abundance, $P_{max}=0.977$.

The high GBDI of leaves did not result from even allocation among pathways. For pathway k, writing $p_{i}=P_{k}q_{i}$ transforms its pre-logarithmic contribution into

$$\sum_{i\in k}\sqrt{p_{i}P_{k}}=P_{k}\sum_{i\in k}\sqrt{q_{i}}=P_{k}\sqrt{D_{\left(1,/2k\right)}},$$

where $D_{\left(1,/2k\right)}$ is the Hill diversity of order one-half within the pathway. Thus, the GBDI term is sensitive to abundance-weighted effective intrapathway diversity, not merely raw terpenoid richness. The leaf profile is consistent with high effective diversification within the dominant terpenoid category, although it does not directly measure enzymatic branching or metabolic flux.

**Figure 4:**
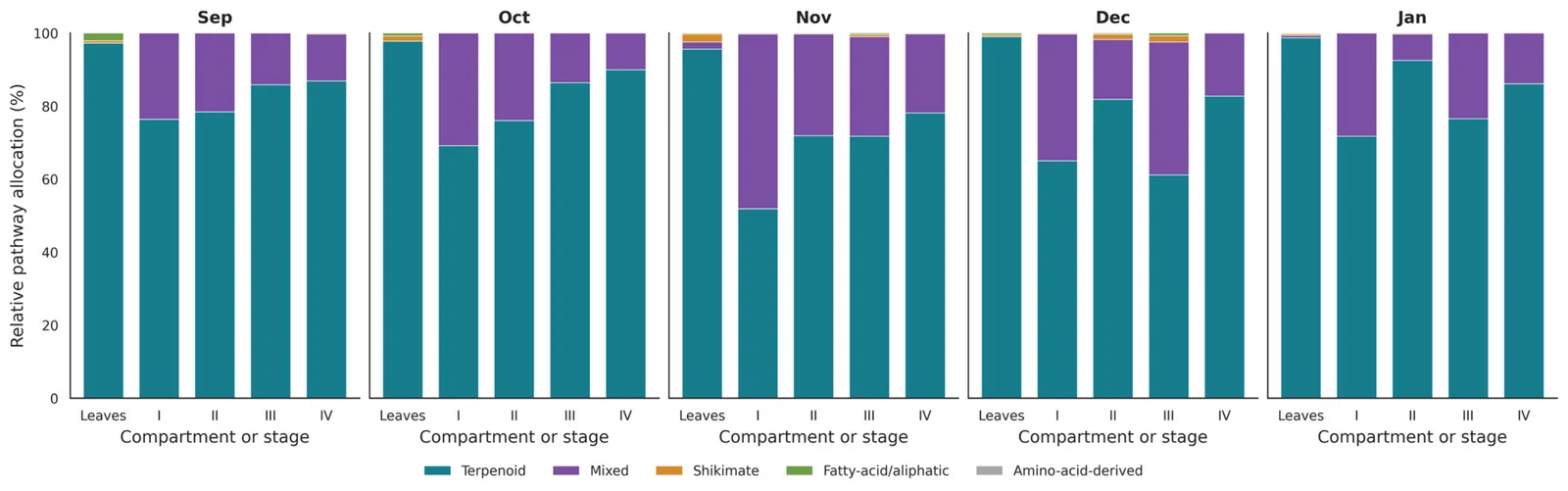
Mean biosynthetic-pathway allocation in leaves and reproductive stages of *Piper mollicomum*. Bars represent normalized pathway abundance averaged across five monthly blocks. The contrast between leaves and reproductive organs demonstrates that high compound-level diversity may occur within one strongly dominant biosynthetic pathway. Sep = September; Oct = October; Nov = November; Dec = December; Jan = January.

Reproductive stages exhibited a different organization. Their total Shannon diversity and GBDI were generally lower than those of leaves, but their pathway entropy was substantially higher. In Stage I, the terpenoid pathway represented a mean of 66.83% of the normalized profile, while the mixed pathway, represented principally by eupatoriochromene, accounted for 33.07% (Table 2, Figure 4). This more balanced allocation generated the highest mean pathway entropy among the evaluated compartments.

The descriptive comparison shows that high compound-level diversity need not coincide with even allocation among annotated pathways. Pathway entropy distinguishes allocation among pathway categories, whereas GBDI combines compound proportions with their assigned pathway totals. Because both metrics depend on the selected classification, their interpretation is conditional on biosynthetic annotation.

✓ High compound diversity associated with extensive branching within one dominant pathway;
✓ Greater balance among pathways associated with lower total compound diversity.

Pathway entropy can distinguish these architectures, while GBDI integrates metabolite diversity and biosynthetic organization into a single compound-sensitive descriptor [8]. For this reason, GBDI should not be interpreted as equivalent to pathway evenness.

### 2.4. Ontogenetic Chemical Reorganization Was Dynamic Rather than Strictly Monotonic

**Figure 5:**
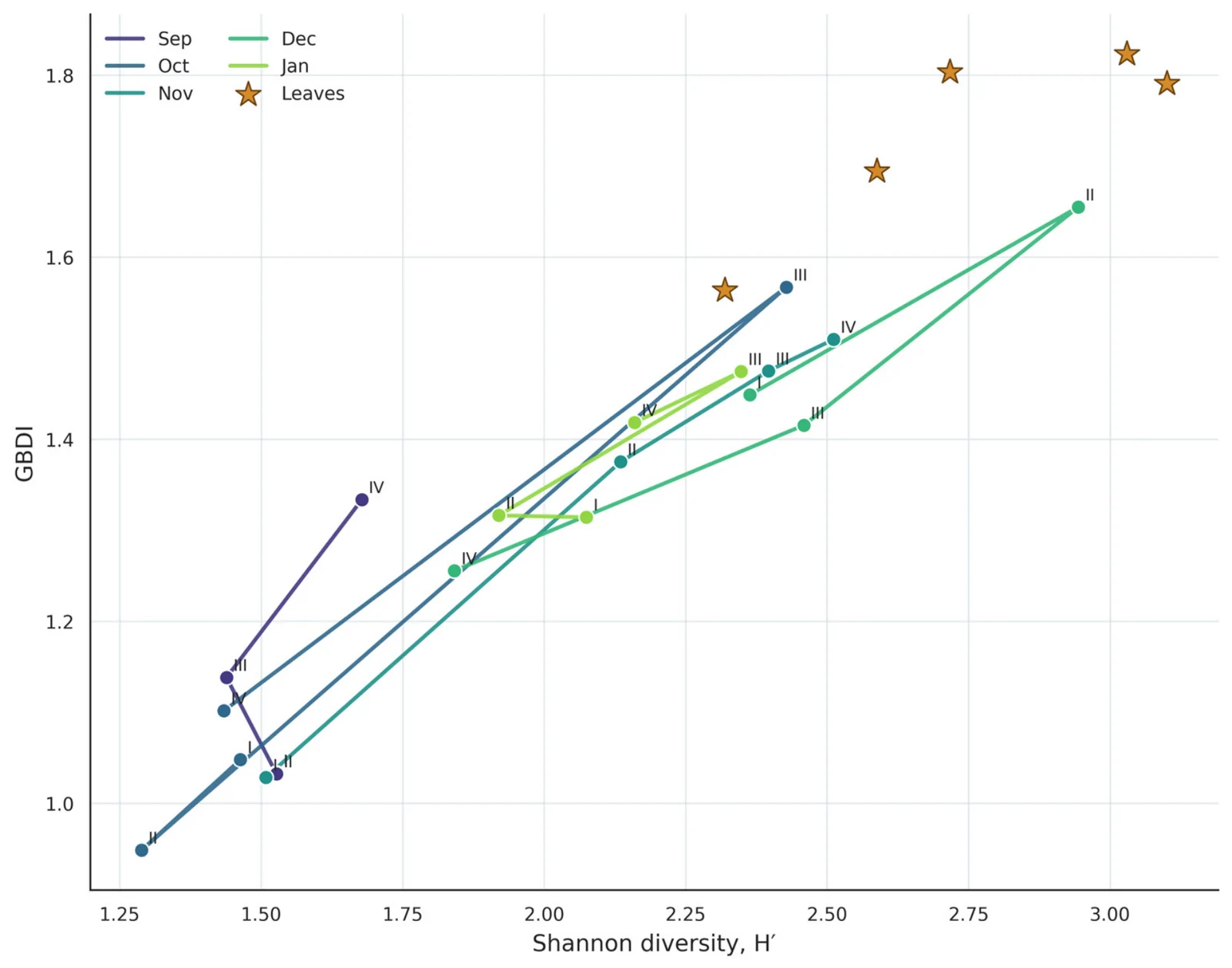
Joint trajectories of Shannon diversity and GBDI during reproductive-organ development. Lines connect Stages I–IV within each monthly block. Leaf profiles are shown as independent vegetative reference points. Differences among trajectories indicate that ontogenetic chemical reorganization was influenced by the monthly context. Sep = September; Oct = October; Nov = November; Dec = December; Jan = January.

The reproductive trajectory did not follow a single monotonic pattern shared by every monthly block (Figure 5). Stage III represented the highest mean of reproductive diversity, but the magnitude and direction of the transitions varied among months.

Ontogenetic variation in plant specialized metabolism is biologically plausible [23,24,25,26], but the present relative profiles do not directly measure production, storage, conversion, emission, or regulatory flux. The observed trajectories are therefore described as ontogenetic compositional variation. Mechanistic explanations involving metabolism, environment, or seasonality remain hypotheses for future experiments.

Stage I generally represented a chemically concentrated initial reproductive state. Its mean dominant-metabolite abundance was 0.388, and it presented the lowest Shannon diversity of the reproductive trajectory. Stage II was associated with a partial redistribution of abundance and greater evenness, with mean Pielou evenness increasing from 0.701 to 0.764.

Stage III had the highest numerical mean Shannon diversity and GBDI among reproductive stages. Exact Shannon decomposition showed that the mean Stage II to III increase of 0.251 nats comprised 0.197 nats (78.3%) from the abundance-weighted within-pathway component and 0.055 nats (21.7%) from the between-pathway component. Neither component differed significantly from zero across five blocks (Wilcoxon *p* = 0.438 and 0.625, respectively), so this is a descriptive decomposition rather than a confirmed mechanistic effect.

The absence of a strictly monotonic trajectory does not invalidate the ordered-game representation. Each transition defines a separate chemical context. In game-theoretical terms, the outcome associated with a strategy depends on the state of the system and the frequencies of the other strategies [9,10,11,12,13]. Similarly, the realized chemical payoff of a metabolite can be positive during one ontogenetic transition and negative during another.

A positive payoff should not be interpreted as a prediction of continued increase or as evidence of greater absolute production. It identifies only a transition-specific relative enrichment in the closed chromatographic profile. Reversals in sign may flag candidate relative-enrichment intervals for subsequent absolute and mechanistic testing.

### 2.5. Realized Chemical Payoffs Quantified Directional Redistribution

For each strategy k, corresponding to a metabolite, chemical class, or biosynthetic pathway, the observed discrete log-ratio change was calculated as:

$$r_{k,t}=ln\left(\frac{p_{k,t+1}}{p_{k,t}}\right)$$

Log-ratio transformations are appropriate for compositional systems because they express changes in one component relative to other components rather than treating proportions as independent variables [32,33,34].

The realized chemical payoff was defined as:

$$g_{k,t}=r_{k,t}-\sum_{j}p_{j,t}r_{j,t}$$

The second term is the initial-abundance-weighted mean log change in the chemical system. Subtracting it produces a centered log-ratio quantity analogous to the relative-performance term of the continuous replicator equation. This is not a formal recovery of a conventional game payoff because no payoff matrix, interaction function, or fitness-generating mechanism is estimated. We therefore define g as a realized chemical payoff only within the operational terminology of Chemical Game Theory.

Conventional compositional data analysis remains the mathematical foundation of the framework. Chemical Game Theory does not claim that centering creates information absent from log-ratios. At a fixed hierarchical level, the difference between two realized payoffs is exactly the change in their pairwise log-ratio,

$$g_{i,t}-g_{l,t}=\ln\left[\frac{p_{i,t+1}/p_{i,t}}{p_{l,t+1}/p_{l,t}}\right],$$

and the abundance-weighted payoffs satisfy the zero-sum identity,

$$\sum_{i}p_{i,t}g_{i,t}=0.$$

The distinction from a conventional centered log-ratio coordinate is the use of the initial-abundance-weighted transition reference rather than an unweighted geometric-mean reference. The substantive contribution is therefore not a replacement for compositional data analysis, but an operational organization of compositional change into ordered state transitions; explicitly defined metabolite-, class-, and pathway-level strategies; aggregation before transformation at each hierarchical level; separation of directional change from abundance-weighted impact; sign consistency across blocks; and joint interpretation with Shannon diversity and GBDI for sample-prioritization hypotheses. This reframing makes the reference, scale, and decision rule explicit while preserving the underlying log-ratio relationships.

Under this definition:

$g_{k,t}>0$ indicates relative chemical expansion;

$g_{k,t}<0$ indicates relative chemical contraction; 

$g_{k,t}\approx 0$ indicates change like the abundance-weighted chemical background. 

These values are context dependent. A positive chemical payoff is not an intrinsic property of a metabolite and does not imply molecular agency, intentional competition, or Darwinian fitness. It indicates that the metabolite or aggregated chemical strategy gained relative representation during a defined transition (Table 3 and Table S4).

Payoff and biological fitness are not synonyms in the present framework. In evolutionary game models, fitness may act as a payoff because reproductive success changes strategy frequencies across generations [11,12,13,14,15,16]. In general game theory, however, a payoff is the outcome or value assigned to a strategy under a defined context and does not intrinsically require reproduction, heritability, or a multigenerational interval [9,10]. Here, realized chemical payoff is a model-defined centered relative change calculated between two measured chemical states. It does not estimate survival, fecundity, inheritance, selection, or persistence across generations. Accordingly, the interpretive combinations in Table 4 describe transition-specific compositional patterns only and are not categories of ecological or Darwinian fitness. We retain the term payoff because it identifies the relative outcome variable in the proposed game-theoretical analogy, always qualified as realized chemical payoff and explicitly delimited from fitness.

#### 2.5.1. Stage I → Stage II

The Stage I to II transition showed an observed directional redistribution between the terpenoid and mixed categories. The terpenoid payoff was positive in all five monthly blocks, with a mean of 0.269 ± 0.191 and mean impact of 0.196. A two-sided exact Wilcoxon signed-rank test did not reach *p* < 0.05 (*p* = 0.0625), so 5/5 denotes descriptive sign consistency rather than confirmatory statistical significance.

**Figure 6:**
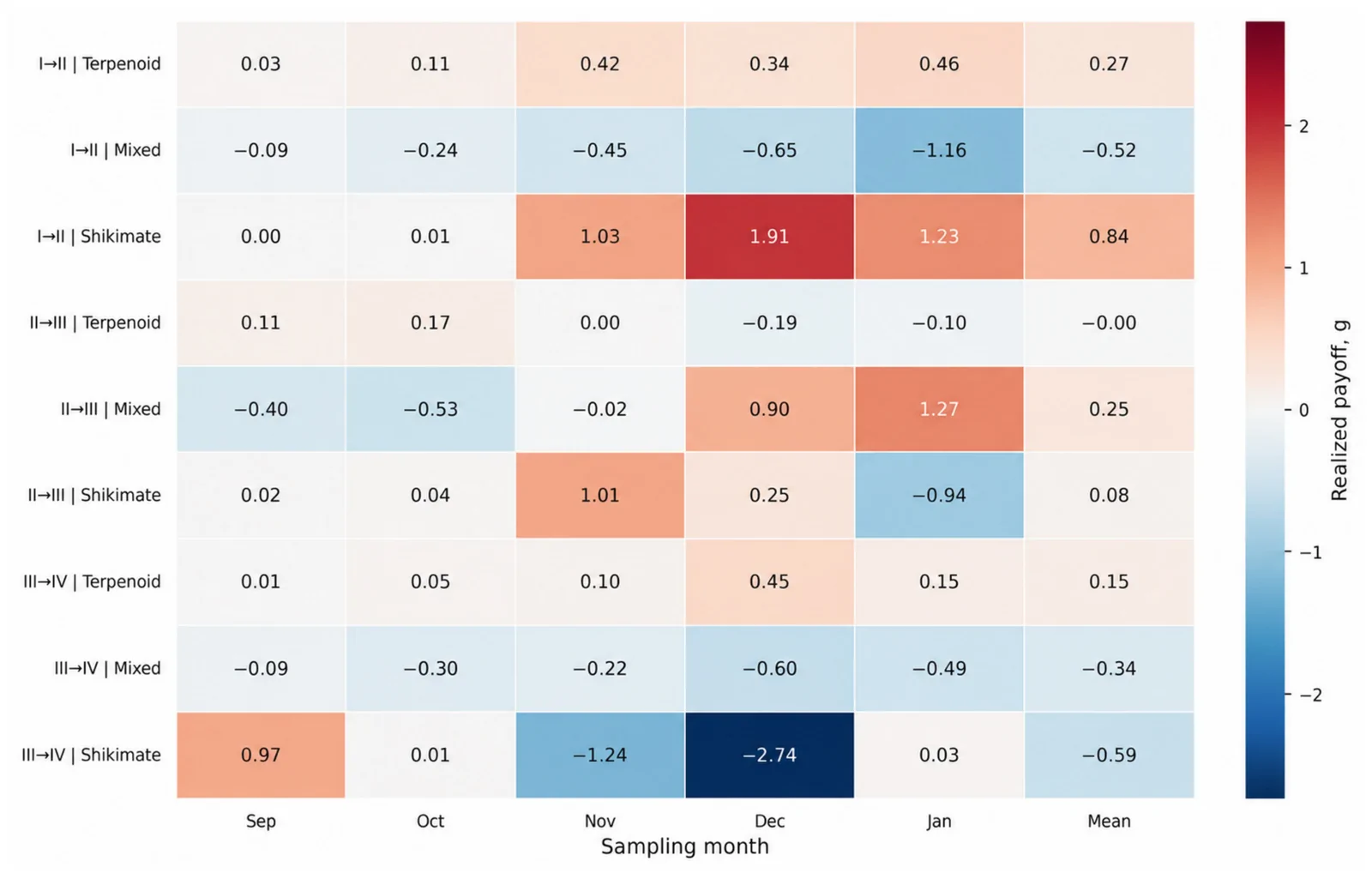
Realized pathway payoffs across reproductive transitions and monthly blocks. Positive values indicate expansion relative to the abundance-weighted chemical background, whereas negative values indicate relative contraction. Payoff is a transition-dependent measure derived from relative-performance principles in evolutionary game theory [9,10,11,12,13], adapted here to closed chromatographic mixtures.

In contrast, the mixed pathway had a consistently negative payoff of −0.518±0.418, with negative values in all five months. Because this pathway represented approximately one-third of the Stage I profile, its negative payoff produced a substantial mean impact of −0.126 (Figure 6).

Within the five observed blocks, this transition was characterized by relative reallocation from a mixed-category-rich initial profile toward greater terpenoid representation. The term reproducible was removed because the pattern has not been replicated in independent populations or sampling series.

The shikimate category exhibited the highest numerical mean payoff, 0.837 ± 0.824, but its impact was only 0.0037 because it occupied a very small fraction of the mixture. This combination is not treated as evidence of biologically important strategic emergence. It is reported as a high proportional change with low quantitative influence and marked sensitivity of magnitude to zero replacement.

#### 2.5.2. Stage II → Stage III

The Stage II → Stage III transition was comparatively neutral at the pathway level. The terpenoid pathway had a mean payoff close to zero, −0.002±0.146, and was positive in three of the five monthly blocks.

The mixed pathway had a positive mean payoff of 0.246 but a large standard deviation of 0.802 and was positive in only two months (Figure 6). Therefore, the positive arithmetic mean did not represent a temporally conserved response.

Exact Shannon decomposition showed that 78.3% of the mean Stage II to III increase arose from the abundance-weighted within-pathway component and 21.7% from the between-pathway component. However, neither component differed significantly from zero across the five blocks. We therefore describe the pattern as predominantly intrapathway in the observed data, without claiming a statistically confirmed mechanism.

This distinction illustrates the importance of hierarchical analysis. A pathway may remain quantitatively stable while metabolites belonging to that pathway undergo substantial replacement.

#### 2.5.3. Stage III → Stage IV

The final transition again showed directional separation between terpenoid and mixed-pathway allocation. The terpenoid pathway presented a positive mean payoff of 0.153±0.176, with positive values in all five months and a mean impact of 0.116.

The mixed pathway exhibited a negative mean payoff of −0.340±0.204, with negative values in every month. Its mean impact was −0.071 (Figure 6). The shikimate pathway also showed a negative mean payoff, but its values were highly variable, and its quantitative contribution was small.

Together with the numerical decline in Shannon diversity and GBDI, the Stage III to IV profiles showed positive terpenoid and negative mixed-category payoffs in all five blocks. The paired difference between these two payoffs was not significant in a two-sided Wilcoxon test (*p* = 0.0625). The pattern is therefore described as directionally consistent within the observed blocks, not as a confirmed pathway-level biological reorganization.

### 2.6. Realized Payoff and Payoff Impact Were Complementary Variables

A central operational distinction of the proposed framework is that payoff and payoff impact answer different descriptive questions. Realized payoff quantifies centered proportional change, whereas payoff impact weights that change by mean relative abundance. A large payoff for a rare component can arise from a small denominator or zero replacement and must not be interpreted as a major system-level change without considering impact and sensitivity.

To distinguish relative directional change from quantitative influence, payoff impact was defined as:

$$I_{k,t}=g_{k,t}\left(\frac{p_{k,t}+p_{k,t+1}}{2}\right)$$

where the term in parentheses is the mean abundance of strategy k across the transition.

Payoff impact is introduced here as a new supporting descriptor. It is not a previously established index in game theory or chemodiversity. Its purpose is to prevent large proportional changes in rare metabolites from being interpreted as the principal drivers of chemical reorganization.

The shikimate pathway during Stage I → II illustrates this distinction. It presented a mean payoff greater than that of the terpenoid pathway, but its impact was approximately 53 times smaller. The shikimate pathway underwent a strong relative increase from a very small initial allocation, while the terpenoid pathway exerted a much greater influence on the total chemical profile.

The four combinations in Table 4 are a conceptual interpretation grid, not a statistical classification used to assign the present observations. No universal High or Low threshold is proposed. In future applications, any threshold must be defined a priori according to analytical precision, biological relevance, and study objectives. In this proof of concept, exact payoff, impact, abundance, sign frequency, and sensitivity values are reported without dichotomizing them.

### 2.7. Compound-Level Dynamics Revealed Ontogenetic Windows Relevant to Bioprospection

Leaves and reproductive stages presented distinct distributions of major constituents (Table 5). Leaves contained a broad terpenoid profile, while the reproductive organ introduced a substantial mixed-pathway contribution associated with eupatoriochromene.

Eupatoriochromene attained its greatest mean abundance in Stage I, representing 33.07% of the normalized profile. Its mean abundance decreased to 19.40% in Stage II, increased moderately to 23.00% in Stage III, and declined again to 15.14% in Stage IV. The highest individual value, 47.86%, occurred in Stage I in November.

Linalool presented an opposite late-development tendency. Its mean abundance increased from 10.12% in Stage I to 19.54% in Stage II, 20.59% in Stage III, and 28.34% in Stage IV. Its highest individual abundance, 56.58%, occurred in Stage IV in September.

Limonene showed predominantly early-stage distribution. Its mean abundance decreased from 11.65% in Stage I to 9.41%, 4.39%, and 2.59% in Stages II, III, and IV, respectively. Its highest individual abundance, 41.83%, occurred in Stage I in September (Table 5).

Natural-product discovery increasingly combines metabolomic profiling, dereplication, chemometric comparison, and biological screening to prioritize extracts and metabolites [27,28,29,30]. The Chemical Game Theory framework adds an ontogenetic and directional dimension to this workflow.

Two distinct criteria can be used for rational collection:

**Target-yield criterion**, selecting the compartment, stage, and month with the greatest abundance of a desired metabolite;

**Strategic-emergence criterion**, selecting a transition in which the metabolite shows a positive realized payoff relative to the chemical background.

The target-yield criterion directly identifies the highest observed relative abundance. The strategic-emergence criterion is an exploratory hypothesis rather than a demonstrated superior bioprospecting strategy. For example, *E,E*-alpha-farnesene in September increased from undetected in Stage III to 1.49% in Stage IV and had a high model-defined payoff but low abundance. This makes it a candidate for follow-up detection or isolation, not evidence of a precursor-product relationship, structural novelty, biological activity, or superiority over a high-abundance target.

A rare compound with a high positive payoff could be overlooked by abundance-only prioritization. Conversely, an abundant metabolite with a payoff near zero may remain quantitatively important without representing an active transition-specific expansion. Therefore, abundance, payoff, and payoff impact should be evaluated together.

**Figure 7:**
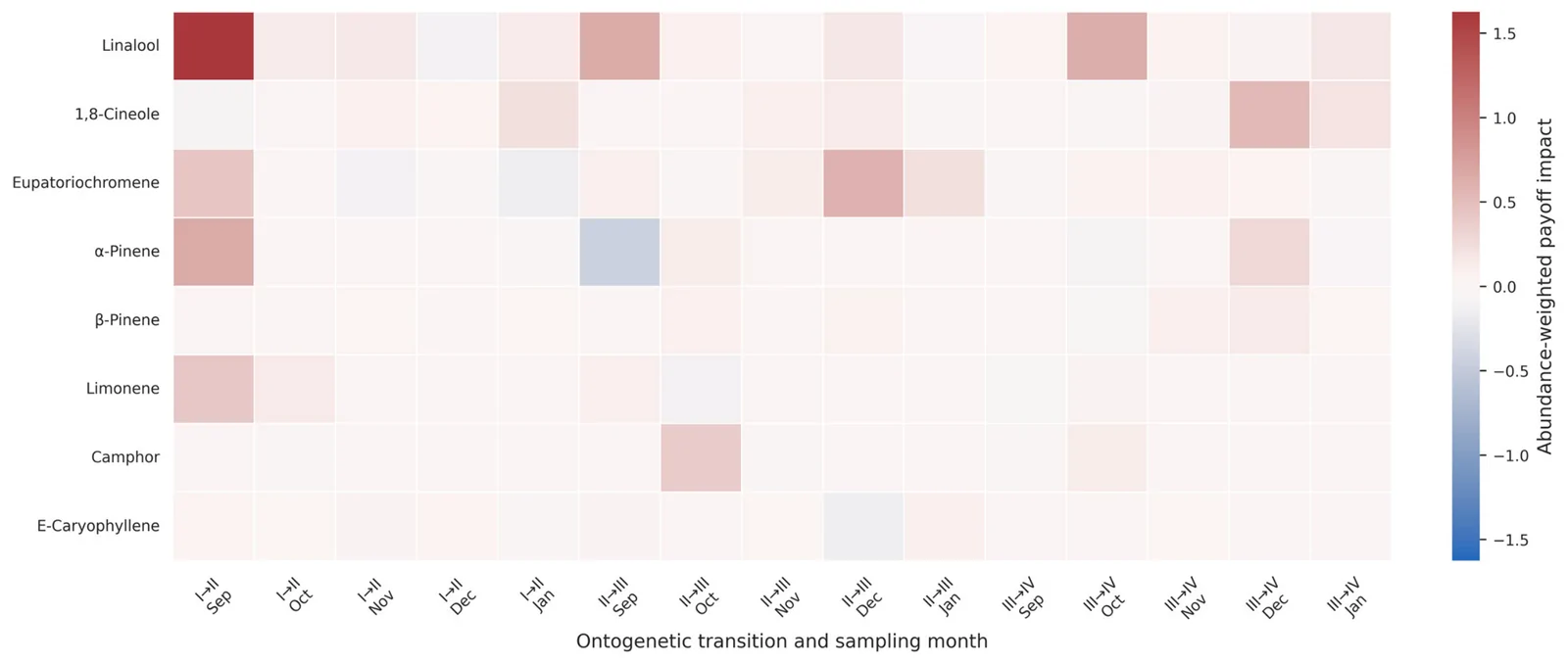
Abundance-weighted realized payoff impacts of selected metabolites during reproductive development. Positive values identify metabolites contributing to relative chemical expansion, and negative values identify compounds contributing to contraction. The figure distinguishes high-payoff rare components from quantitatively influential metabolites.

This strategy complements contemporary metabolomics-guided bioprospection [27,28,29,30], including the dereplication and deep-annotation approaches discussed by Wolfender et al. [29]. It does not replace biological screening. Rather, it provides an additional criterion for deciding which organ, developmental stage, or sampling period should be prioritized for extraction and testing (Figure 7).

### 2.8. Chemical Payoffs Were Dependent on the Hierarchical Level of Analysis

The same ontogenetic transition produced different results when evaluated at metabolite, chemical-class, and biosynthetic-pathway levels. This scale dependence is expected because plant specialized metabolism is hierarchically organized [23,24,25,26].

At the metabolite level, payoff identifies individual compounds gaining or losing relative representation. At the chemical-class level, it evaluates the collective dynamics of structurally related compounds. At the pathway level, it describes the redistribution of total chemical investment among broader biosynthetic architectures.

Pathway-level payoffs are calculated after aggregation and therefore need not equal the sum or mean of constituent payoffs. For the September Stage III to IV transition, the terpenoid category increased from 0.8583 to 0.8690. After zero replacement and reclosure, its aggregate log change was 0.01331, the system reference was −0.00131, and its pathway payoff was 0.01462. By contrast, the unweighted mean of the metabolite-level terpenoid payoffs was 0.33842. This numerical example demonstrates the non-commutativity of aggregation and logarithmic transformation.

$$ln\left(\frac{\sum_{i}p_{i,t+1}}{\sum_{i}p_{i,t}}\right)\neq \sum_{i}ln\left(\frac{p_{i,t+1}}{p_{i,t}}\right)$$

This mathematical property explains how the terpenoid pathway could exhibit a mean payoff close to zero during Stage II → III while individual terpenoids underwent substantial positive and negative changes.

Scale dependence is a mathematical consequence of aggregating the compositional vector before logarithmic transformation. It is not a computational inconsistency, but neither does it prove distinct causal levels of biological organization. Biological interpretation of metabolite, class, and pathway levels depends on independently justified annotations and ideally on enzymatic, genomic, isotopic, or flux evidence.

### 2.9. Treatment of Zeros and Sensitivity of Realized Payoffs

Zero values require particular attention because log-ratio calculations are undefined when one or more components equal zero [32,33,34,35,36]. In chromatographic datasets, a zero may represent true absence, concentration below the analytical detection limit, or failure to report a very low-abundance component (Table S5; Figure S2).

The principal analysis used one-half of the smallest positive value, and one-tenth was evaluated as a deliberately smaller sensitivity condition. These factors bracketed two plausible substitutions but were not selected as uniquely optimal estimators. Alternative multiplicative replacement and model-based imputation methods exist [35,36].

Replacement of zeros is an established practical requirement in conventional log-ratio analysis, although the selected replacement rule can influence estimates for rare components [35,36]. For this reason, directional stability was evaluated by comparing payoff signs under the two replacement conditions.

Across 1935 metabolite-, class-, and pathway-level comparisons, 99.64% retained the same sign, and payoff magnitudes were strongly correlated between treatments (Pearson r = 0.989). However, the median absolute difference was 0.162, and only 34.11% differed by no more than 0.10. Therefore, payoff direction was robust within the tested range, whereas magnitude, especially for zero-involving transitions, was sensitive to the replacement rule.

The greatest numerical sensitivity occurred for strategies that appeared or disappeared between adjacent stages. Payoff magnitudes for these components should be interpreted cautiously, even when their direction was stable. Reporting payoff impact and initial and final abundances reduces the risk of overemphasizing large proportional changes in trace constituents.

### 2.10. Scope and Limitations of the Proof of Concept

The present results were derived from relative chromatographic abundance. A relative increase may reflect an absolute increase in the component, an absolute decrease in other components, or both. Without internal standards, absolute quantification, essential-oil yield integrated with compound abundance, and tissue biomass, the framework cannot identify increased production or biosynthetic flux. Accordingly, the results are interpreted as compositional reorganization and relative enrichment rather than redistribution of biosynthetic investment.

The empirical domain of this proof of concept is intentionally restricted to plant specialized metabolites represented in essential oils, which are directly relevant to natural-product chemistry and bioprospecting. Primary metabolism supplies carbon skeletons, energy, reducing equivalents, and precursors to specialized metabolism [1,2,3,24,25], but primary metabolites were not measured in the source study and cannot be reconstructed from the essential-oil profiles. Adding unobserved primary-metabolic variables would therefore introduce unsupported information rather than make the present analysis more complete. The mathematical framework could be applied to primary-metabolite data when those compounds are measured through an analytically appropriate and internally comparable platform, but the present results do not model nutrient status, central metabolic flux, disease state, biomass production, agricultural yield, or optimum cultivation conditions. The proposed candidate collection windows refer only to the relative composition of the measured specialized-metabolite fraction.

This distinction follows directly from the compositional nature of the data [32,33,34]. The abundance of one component may increase proportionally because that compound increased in absolute concentration, because other compounds decreased, or through a combination of both processes.

Future applications should combine relative profiles with internal standards, absolute quantification, tissue biomass, and essential-oil yield. Such measurements would allow relative chemical expansion to be distinguished from absolute metabolite production.

Chromatographic peak-area percentages are method-dependent, response-weighted signals. In the source dataset, GC–MS was used for constituent identification and GC–FID for relative quantification, an established hyphenated strategy for profiling complex essential oils containing many constituents [31]. Such profiling does not, however, substitute for calibration when absolute mass or molar quantities are required. FID response is broadly related to combustible carbon, but it varies with molecular formula and functional groups, and the assumption that all relative response factors equal unity can introduce compound-dependent and class-dependent bias [47]. Because authentic standards or experimentally determined response factors were not available for every constituent in the published dataset, the present proportions must be interpreted as semiquantitative relative chromatographic composition, not as calibrated mass fractions or molar fractions. No retrospective universal correction factor would be scientifically defensible. Under a common extraction and analytical protocol, the response coefficient of a given compound is expected to remain approximately constant, so it cancels from the unclosed same-compound area ratio between states. Nevertheless, detector response still affects compositional closure, abundance weighting, aggregation, and comparisons among chemical classes or pathways. Consequently, between-pathway payoff magnitudes are interpreted cautiously. Future prospective applications should use an internal standard together with experimental or validated predicted relative response factors, compound-specific calibration when feasible, and independent measurements of essential-oil yield and tissue biomass.

The five sampling months were used as temporal blocks and do not represent independent populations or experimental replicates. The inferential analyses are exploratory. Terms such as reproducible, recurrent, and significant compartmental difference have been restricted to results supported by the corresponding test. Statements based on 5/5 directional agreement are explicitly labeled descriptive sign consistency.

The empirical evaluation is limited to one species and one essential-oil dataset. The five organs or developmental states and monthly blocks provide multiple compositional contexts within that dataset, but they are not independent chemical systems and do not establish external validity across chemotypes, taxa, matrices, or analytical platforms. Accordingly, the present study is designated a proof of concept. Evaluating monoterpene-dominated, sesquiterpene-dominated, phenylpropanoid-rich, and chemically broader datasets is an appropriate next validation stage. However, combining literature datasets retrospectively would confound biological composition with differences in extraction, plant material, chromatographic columns, detector response, reporting thresholds, constituent identification, and zero prevalence. Such validation should therefore use prospectively harmonized datasets or independently analyzed datasets with explicit analytical comparability, rather than adding heterogeneous examples that could give a misleading impression of general validation.

Future studies should include biological replicates, additional populations, controlled environmental treatments, and, when possible, longitudinal sampling. Experimental manipulation of developmental or environmental conditions would also help determine whether recurrent payoff patterns correspond to regulated biosynthetic changes.

Most importantly, the proposed framework does not demonstrate competition, cooperation, intentional behavior, selection, biosynthetic flux, or causal interaction among metabolites. Its terminology is an operational analogy based on constrained allocation and centered relative change. Demonstrated conclusions are limited to mathematical properties of the descriptors and descriptive patterns in the analyzed *P. mollicomum* profiles. Mechanistic interpretation, cross-platform transferability, and bioprospecting performance remain hypotheses for prospective validation.

### 2.11. General Implications for Chemodiversity and Rational Bioprospection

The joint use of Shannon diversity, GBDI, pathway entropy, realized payoff, and payoff impact produces a multidimensional description of plant chemical organization.

Shannon diversity identifies chemically broad and evenly distributed mixtures [2,3,4,5]. GBDI evaluates compound diversity in relation to biosynthetic architecture [8]. Pathway entropy distinguishes balanced route allocation from diversification within a dominant pathway. Realized payoff identifies transition-dependent chemical expansion or contraction. Payoff impact estimates the quantitative influence of that directional change (Figure S3).

These descriptors can support a sequential bioprospecting workflow:

compartment selection→developmental-stage selection→chemodiversity analysis→payoff analysis→target prioritization→biological screening

A compartment with high Shannon diversity may be prioritized for broad untargeted screening. A state with high GBDI may provide access to a more extensively expressed biosynthetic architecture. A high-payoff, low-impact metabolite may represent a rare emerging target. A high-payoff, high-impact metabolite may combine developmental enrichment with practical extractive relevance.

In *P. mollicomum*, leaves represented a highly diversified but predominantly terpenoid chemical compartment. The reproductive organs introduced a substantial mixed-pathway contribution, associated primarily with eupatoriochromene. Early reproductive development favored this mixed-pathway-rich organization, whereas late development was associated with increased terpenoid representation. These differences could not be fully summarized by a single diversity index. Leaves displayed high Shannon diversity and GBDI but low pathway entropy. Early reproductive stages displayed lower total compound diversity but greater interpathway balance. Realized payoffs subsequently revealed the directions in which this biosynthetic allocation changed during development.

Chemical Game Theory provides an operational link between descriptive phytochemical profiling and hypothesis generation for sample prioritization. It reports relative compositional change, its hierarchical dependence, and its abundance-weighted impact. It does not determine absolute production, enzymatic regulation, metabolic flux, biological activity, or causal interaction.

The *P. mollicomum* dataset demonstrates the calculation and interpretation of the proposed descriptors in one essential-oil case study. Broader applications to seasonal, circadian, stress-induced, cultivation, domestication, post-harvest, and LC-MS datasets are mathematically possible but remain unvalidated. Each application will require appropriate replication, preprocessing, annotation, zero treatment, and, where biological production is inferred, absolute quantification.

## 3. Materials and Methods

### 3.1. Study Design and Source of the Chemical Dataset

The present study constitutes a secondary quantitative analysis of a previously published essential-oil dataset obtained from leaves and four developmental stages of the reproductive organ of *Piper mollicomum* Kunth [31]. No new plant collections, essential-oil extractions, or chromatographic analyses were performed specifically for the present investigation.

The botanical identification, collection site, sampling design, reproductive-stage characterization, essential-oil extraction, GC–MS conditions, constituent identification, and calculation of relative chromatographic abundances were described in the original study [31]. Briefly, the dataset comprised leaf and reproductive-organ essential-oil profiles obtained monthly from September 2020 to January 2021. Reproductive organs were separated into four ordered developmental stages, designated Stage I, Stage II, Stage III, and Stage IV.

Only the chemical-composition data were analyzed in the present study. Data concerning floral visitors or other plant–animal interactions reported in the original publication were not included because the objective was specifically to formulate and demonstrate Chemical Game Theory as a tool for interpreting dynamic chemodiversity.

The experimental design contained five monthly blocks:

b=1,2,…,5

and five compartments or chemical states within each block:

c=1,2,…,5

corresponding to leaves and reproductive Stages I–IV.

Leaves were treated as a vegetative reference compartment and were not considered an ontogenetic Stage 0. Leaves and reproductive organs are biologically distinct compartments, and a direct leaf-to-reproductive-stage payoff would combine organ identity and ontogenetic progression.

Realized ontogenetic payoffs were therefore calculated only for the following adjacent transitions:

Stage I→Stage IIStage II→Stage IIIStage III→Stage IV

Each transition was calculated separately within each monthly block. Thus, Stage I collected in September was compared with Stage II collected in September, rather than with a reproductive stage collected in another month. This blocked approach reduced the confounding of developmental reorganization with temporal variation. All data are presented in Supplementary File S1 of this article.

### 3.2. General Applicability to GC-MS and LC-MS Data

Although the equations can be applied mathematically to any nonnegative, aligned abundance matrix, empirical validation in this study is restricted to GC-MS and GC-FID essential-oil profiles. Application to LC-MS remains proposed, not demonstrated. LC-MS datasets with many features and missing values require platform-specific preprocessing, missingness assessment, zero handling, annotation uncertainty analysis, and independent validation before the behavior of the framework can be generalized.

The minimum input consists of a chemical-abundance matrix:

$$X=\left[x_{i,s}\right]$$

where $x_{i,s}$ is the measured abundance of chemical features or metabolites i in chemical state s.

The index i represents metabolites or reproducible chromatographic features:

i=1,2,…,S

and s represents samples, compartments, treatments, time points, or developmental states:

s=1,2,…,N

For GC–MS data, $x_{i,s}$ may represent integrated peak area, normalized total-ion-current area, or the reported relative percentage of an identified volatile constituent. For LC–MS data, $x_{i,s}$ may represent the integrated area or intensity of a reproducible mass-spectrometric feature defined by its mass-to-charge ratio and retention time.

For pathway-level GBDI and Chemical Game Theory calculations, chemical features should preferably be annotated to a level that permits assignment to a biosynthetic pathway. Unidentified features may still be included in Shannon diversity and metabolite-level payoff calculations, if they are reproducibly aligned among samples. However, they cannot contribute to pathway-sensitive indices unless they are assigned to an explicit unclassified category.

The following information is recommended for each chromatographic feature:

1. Unique feature or compound identifier;
2. Metabolite name or feature label;
3. Retention time;
4. Mass-spectral information;
5. Measured abundance in every sample;
6. Chemical class;
7. Biosynthetic-pathway assignment;
8. Identification or annotation confidence.

For untargeted LC–MS applications, feature alignment, blank subtraction, signal filtering, adduct grouping, isotope removal, and normalization should be completed before the Chemical Game Theory analysis. These preprocessing procedures are platform dependent and should be described according to the analytical workflow employed in each study.

### 3.3. Extraction and Curation of the Piper mollicomum Composition Matrix

The complete individual chemical profiles reported as “% in 1” in Supplementary Table S3 of the original publication were transcribed into a structured data matrix [31]. These profiles were selected because they represented complete and internally reconcilable constituent lists for each chemical state (Supplementary File S1).

Tables containing only averages of selected major constituents were used for descriptive verification but were not employed as compositional input because they did not contain every detected constituent and therefore did not constitute closed chemical profiles.

The following information was extracted for each chromatographic record: compound name; calculated retention index; literature retention index; identification method; reported relative abundance; sampling month; plant compartment or reproductive stage. 

Compound names were harmonized before analysis to prevent differences in capitalization, spacing, stereochemical notation, or typography from generating duplicate chemical players. Synonymous entries were consolidated when they clearly represented the same compound.

All abundance values were required to satisfy:

$$x_{i,s}\geq 0.$$

If a negative abundance produced during data preprocessing had occurred, it would have been replaced by zero according to:

$$x_{i,s}^{+}=max\left(x_{i,s},0\right).$$

No additional abundance threshold was applied to the calculation of Shannon diversity, GBDI, or compound-level payoff. Therefore, every reported constituent was retained in the principal chemical-state calculations.

For graphical visualization of compound-level payoffs, a reduced set of metabolites was selected to preserve figure readability. A compound was retained in the graphical subset when its maximum abundance among reproductive profiles was at least 0.02:

$$max\left(p_{i,s}\right)\geq 0.02$$

or when it was detected in at least five reproductive profiles:

$$\sum_{s=1}^{N}1\left(p_{i,s}>0\right)\geq 5$$

The complete compound-level calculations, without graphical filtering, were retained in the Supplementary Materials.

### 3.4. Biosynthetic-Pathway and Chemical-Class Assignment

Each identified constituent was assigned to a principal biosynthetic pathway and a chemical structural class. Assignments were based on the reported compound identity and established biosynthetic relationships of plant specialized metabolites [23,24,25,26].

The principal pathway categories were: terpenoid; shikimate; mixed biosynthetic origin; fatty-acid/aliphatic; amino-acid-derived. 

Terpenes and terpenoids were assigned to the terpenoid pathway regardless of whether their immediate precursors originated predominantly from the mevalonate or methylerythritol phosphate routes. These routes could be analyzed separately in future studies when sufficient enzymatic, genomic, isotopic, or structural information is available.

Eupatoriochromene was assigned to a mixed biosynthetic pathway because its carbon skeleton combines units of distinct biosynthetic origin. Benzyl benzoate was assigned to the shikimate pathway because its aromatic moieties arise from phenylalanine-derived benzenoid metabolism.

The chemical structural classes used in the analysis were: monoterpene hydrocarbons; oxygenated monoterpenes; sesquiterpene hydrocarbons; oxygenated sesquiterpenes; oxygenated diterpenes; mixed chromenes; shikimate-derived benzenoids; aliphatic derivatives; nitrogenous derivatives. 

Assignments are provided at an auditable compound-level table in Supplementary File S1. For every detected metabolite, the table reports the original and canonical names, chemical class, biosynthetic category, assignment basis, and supporting reference group. Pathway classification affects GBDI, pathway entropy, and pathway-level payoffs but does not affect compound-level Shannon diversity.

### 3.5. Compositional Closure and Within-State Normalization

Chromatographic relative peak areas are compositional because their total is constrained and the values represent relative rather than independent absolute quantities [32,33,34]. Each chemical state was therefore normalized by compositional closure.

For chemical state s, the total reported abundance was calculated as:

(1) $$T_{s}=\sum_{i=1}^{S_{s}}x_{i,s}$$

where $T_{s}$ is the total abundance of the constituents reported in state s, $x_{i,s}$ is the original abundance of compound i, and $S_{s}$ is the number of reported compounds.

The closed relative abundance of compound i was then:

(2) $$p_{i,s}=\frac{x_{i,s}}{T_{s}}$$

Substituting the definition of $T_{s}$:

(3) $$p_{i,s}=\frac{x_{i,s}}{\sum_{j=1}^{S_{s}}x_{j,s}}$$

The resulting relative abundances satisfy:

(4) $$0\leq p_{i,s}\leq 1$$

and:

(5) $$\sum_{i=1}^{S_{s}}p_{i,s}=1$$

This normalization ensured comparability among profiles whose reported identified constituents did not sum exactly to 100%.

The closure operation does not transform relative data into absolute concentrations. It expresses every compound as a fraction of the total identified chemical profile. Consequently, all subsequent diversity indices and realized payoffs describe relative chemical organization.

### 3.6. Compound-Level Chemodiversity Descriptors

#### 3.6.1. Chemical Richness

Chemical richness was defined as the number of compounds with positive abundance in state s:

(6) $$S_{s}=\sum_{i=1}^{S}1\left(p_{i,s}>0\right)$$

where 1 is an indicator function equal to 1 when the condition is true and 0 when the condition is false.

#### 3.6.2. Shannon Diversity

Shannon diversity was calculated as:

(7) $$H_{s}^{\prime }=-\sum_{i=1}^{S_{s}}p_{i,s}lnp_{i,s}$$

where $p_{i,s}$ is the normalized abundance of compound i in chemical state s.

Compounds with zero abundance were omitted from the summation because:

(8) $$\underset{p\to 0^{+}}{lim}plnp=0$$

Shannon diversity increases when a chemical profile contains more constituents and when abundance is distributed more evenly among them [2,3,4,5].

#### 3.6.3. Pielou Evenness

Pielou evenness was calculated as:

(9) $$J_{s}=\frac{H_{s}^{\prime }}{\ln S_{s}}$$

for:

(10) $$S_{s}>1$$

Pielou evenness approaches 1 when abundance is distributed uniformly among the detected compounds.

#### 3.6.4. Hill Effective Diversities

Hill diversity of order 1 was calculated as:

(11) Ds  1=expHs′

Hill diversity of order 2 was calculated as:

(12) Ds  2=1∑i=1Sspi,s 2

These quantities express diversity as the effective number of equally abundant compounds [4,5].

#### 3.6.5. Compound Dominance

The abundance of the dominant compound was calculated as:

(13) $$p_{max,s}=\underset{1\leq i\leq S_{s}}{max}p_{i,s}$$

The combined abundance of the three most abundant compounds was calculated as:

(14) $${Top3}_{s}=p_{(1),s}+p_{(2),s}+p_{(3),s}$$

where:

$$p_{(1),s}\geq p_{(2),s}\geq p_{(3),s}$$

are the three greatest relative abundances in state s.

### 3.7. Biosynthetic-Pathway Descriptors

Let k denote a biosynthetic pathway:

$$k=1,2,\ldots ,K_{s}$$

The total relative abundance assigned to pathway k in chemical state s was:

(15) $$P_{k,s}=\sum_{i\in k}p_{i,s}$$

Because the compound profile was closed:

(16) $$\sum_{k=1}^{K_{s}}P_{k,s}=1$$

Pathway richness was defined as:

(17) $$K_{s}=\sum_{k=1}^{K}1\left(P_{k,s}>0\right)$$

Pathway entropy was calculated as:

(18) $$H_{path,s}=-\sum_{k=1}^{K_{s}}P_{k,s}lnP_{k,s}$$

The effective number of biosynthetic pathways was:

(19) Dpath,s  1=expHpath,s

The abundance of the dominant pathway was:

(20) $$P_{max,s}=\underset{1\leq k\leq K_{s}}{max}P_{k,s}$$

Pathway entropy and dominant-pathway abundance were used to distinguish even allocation among pathways from compound diversification occurring within one dominant pathway.

For descriptive analysis of intrapathway diversity, the abundance of compound i conditional on its membership in pathway k was:

(21) $$q_{i|k,s}=\frac{p_{i,s}}{P_{k,s}}$$

Within each pathway:

(22) $$\sum_{i\in k}q_{i|k,s}=1$$

The effective compound diversity within pathway k was:

(23) Dwithin,k,s  1=exp−∑i∈kqi∣k,slnqi∣k,s

This descriptor measures intrapathway diversification independently of the total abundance assigned to the pathway.

### 3.8. General Biosynthetic Diversity Index

GBDI was calculated according to the previously proposed formulation [8]:

(24) $${GBDI}_{s}=ln\left[1+\sum_{i=1}^{S_{s}}{\left(p_{i,s}P_{k(i),s}\right)}^{\alpha }\right]$$

where $p_{i,s}$ is the normalized abundance of compound i, $P_{k(i),s}$ is the total abundance of the biosynthetic pathway containing compound i, and k(i) identifies the pathway assigned to compound i.

The abundance-sensitivity exponent was fixed at:

(25) α=0.5

following the original GBDI formulation [8]. The square-root weighting reduces excessive dominance by highly abundant compounds while retaining contributions from quantitatively relevant minor constituents.

The contribution of pathway k to the pre-logarithmic GBDI term was:

(26) $$C_{k,s}=\sum_{i\in k}{\left(p_{i,s}P_{k,s}\right)}^{\alpha }$$

Consequently:

(27) $${GBDI}_{s}=ln\left(1+\sum_{k=1}^{K_{s}}C_{k,s}\right)$$

GBDI and pathway entropy are not equivalent. Pathway entropy describes the distribution of abundance among pathways, whereas GBDI remains sensitive to the compound-level organization within those pathways [8].

### 3.9. Game-Theoretical Representation of the Chemical System

In the proposed Chemical Game Theory framework, metabolites constitute elementary chemical players or strategies; chemical classes constitute intermediate aggregated strategies; biosynthetic pathways constitute higher-order aggregated strategies; normalized abundances represent realized strategy frequencies or allocations; reproductive stages represent ordered chemical states; and changes between adjacent stages represent chemical-game transitions. 

The term “player” does not imply consciousness, intentionality, or autonomous decision-making. It identifies a chemical component whose relative representation changes within a constrained mixture.

The framework was derived from the relative-performance logic of evolutionary games and replicator dynamics, in which the success of a strategy is measured relative to the average performance of the population [9,10,11,12,13]. Chemical Game Theory transfers this logic to chromatographic abundance.

The game was calculated at three hierarchical levels:

L=1, 2, 3

corresponding to metabolite, chemical class, and biosynthetic pathway.

At the metabolite level:

(28) $$z_{k,t}=p_{i,t}$$

At the chemical-class level:

(29) $$z_{k,t}=\sum_{i\in C_{k}}p_{i,t}$$

where $C_{k}$ is the set of metabolites belonging to chemical class k.

At the biosynthetic-pathway level:

(30) $$z_{k,t}=P_{k,t}=\sum_{i\in B_{k}}p_{i,t}$$

where $B_{k}$ is the set of metabolites assigned to biosynthetic pathway k.

The symbol $z_{k,t}$ therefore denotes the abundance of strategy k at the analytical hierarchy being evaluated. This notation prevents confusion between individual-metabolite abundance $p_{i,t}$ and aggregated class or pathway abundance.

Aggregation was performed before log-growth calculation. Therefore, the payoff of an aggregated pathway is not the arithmetic mean or sum of the payoffs of its constituent metabolites.

### 3.10. Treatment of Zeros

The diversity indices were calculated directly from the normalized chemical profiles, with zero-abundance terms omitted from logarithmic entropy expressions. Realized-payoff calculations required an explicit treatment of zeros because ln 0 is undefined [32,33,34,35,36].

For analytical hierarchy L, the smallest observed positive abundance was:

(31) $$m_{L}^{+}=min\left\{z_{k,t}:z_{k,t}>0\right\}$$

For the principal analysis, the replacement value was:

(32) $$\delta_{L}^{\left(0.5\right)}=0.5m_{L}^{+}$$

The zero-replaced abundance was defined as:

(33a) $${\tilde{z}}_{k,t}=z_{k,t}\;\mathrm{when}\;z_{k,t}>0$$

and:

(33b) $${\tilde{z}}_{k,t}=\delta_{L}^{\left(0.5\right)}\;\mathrm{when}\;z_{k,t}=0$$

After zero replacement, the strategy vector was reclosed:

(34) $$z_{k,t}^{*}=\frac{{\tilde{z}}_{k,t}}{\sum_{j=1}^{K_{L}}{\tilde{z}}_{j,t}}$$

Therefore:

(35) $$\sum_{k=1}^{K_{L}}z_{k,t}^{*}=1$$

where $K_{L}$ is the number of strategies at hierarchy L.

For sensitivity analysis, a smaller replacement value was used:

(36) $$\delta_{L}^{\left(0.1\right)}=0.1m_{L}^{+}$$

followed by the same reclosure procedure.

Zero replacement is necessary for conventional log-ratio analysis but can influence estimates for rare components [35,36]. For this reason, payoff direction was compared under the two replacement conditions.

### 3.11. Stepwise Derivation of Realized Chemical Payoff

#### 3.11.1. Transition-Specific Log Growth

For strategy k, changing from state t to adjacent state t+1, compositional log growth was calculated as:

(37) $$r_{k,t}=ln\left(\frac{z_{k,t+1}^{*}}{z_{k,t}^{*}}\right)$$

A positive value $r_{k,t}>0$ indicates an increase in relative abundance, whereas $r_{k,t}<0$ indicates a decrease in relative abundance.

However, log growth alone is not yet the realized payoff because it does not account for the change experienced by the entire chemical system.

#### 3.11.2. Abundance-Weighted Chemical Background

The abundance-weighted mean log growth of the system was calculated as:

(38) $${\bar{r}}_{t}=\sum_{j=1}^{K_{L}}z_{j,t}^{*}r_{j,t}$$

where $z_{j,t}^{*}$ is the initial zero-treated and reclosed abundance of strategy j, and $r_{j,t}$ is its transition-specific log growth. Substituting Equation (37):

(39) $${\bar{r}}_{t}=\sum_{j=1}^{K_{L}}z_{j,t}^{*}ln\left(\frac{z_{j,t+1}^{*}}{z_{j,t}^{*}}\right)$$

This term plays a role analogous to mean population performance in evolutionary game models [9,10,11,12,13].

#### 3.11.3. Realized Chemical Payoff

The realized chemical payoff of strategy k was defined as:

(40) $$g_{k,t}=r_{k,t}-{\bar{r}}_{t}$$

Substituting Equations (37) and (39):

(41) $$g_{k,t}=ln\left(\frac{z_{k,t+1}^{*}}{z_{k,t}^{*}}\right)-\sum_{j=1}^{K_{L}}z_{j,t}^{*}ln\left(\frac{z_{j,t+1}^{*}}{z_{j,t}^{*}}\right)$$

A positive payoff $g_{k,t}>0$ indicates that strategy k expanded more rapidly than the abundance-weighted chemical background.

A negative payoff $g_{k,t}<0$ indicates relative chemical contraction.

A payoff close to zero $g_{k,t}\approx 0$ indicates that the strategy changed at approximately the same rate as the abundance-weighted chemical system.

Because the realized payoff is centered:

(42) $$\sum_{k=1}^{K_{L}}z_{k,t}^{*}g_{k,t}=0$$

To demonstrate this property, Equation (40) can be substituted into the weighted sum:

(43) $$\sum_{k=1}^{K_{L}}z_{k,t}^{*}g_{k,t}=\sum_{k=1}^{K_{L}}z_{k,t}^{*}\left(r_{k,t}-{\bar{r}}_{t}\right)$$

Expanding the expression:

(44) $$\sum_{k=1}^{K_{L}}z_{k,t}^{*}g_{k,t}=\sum_{k=1}^{K_{L}}z_{k,t}^{*}r_{k,t}-{\bar{r}}_{t}\sum_{k=1}^{K_{L}}z_{k,t}^{*}$$

Because:

$$\sum_{k=1}^{K_{L}}z_{k,t}^{*}=1$$

and:

$$\sum_{k=1}^{K_{L}}z_{k,t}^{*}r_{k,t}={\bar{r}}_{t}$$

it follows that:

(45) $$\sum_{k=1}^{K_{L}}z_{k,t}^{*}g_{k,t}={\bar{r}}_{t}-{\bar{r}}_{t}=0$$

This zero-centered property is fundamental to the proposed framework. Positive relative gains must be balanced by relative losses elsewhere in the closed chemical system.

The expression realized chemical payoff is introduced in this study as an operational name for the centered log-ratio quantity. It is inspired by relative-performance reasoning in replicator dynamics [9,10,11,12,13], but it is not a conventional payoff inferred from a payoff matrix and does not represent Darwinian fitness, interaction strength, intentional competition, or autonomous molecular behavior.

### 3.12. Appearance, Persistence, and Disappearance of Chemical Strategies

Each chemical strategy was classified according to its observed abundance before zero replacement.

A persistent strategy satisfied:

(46) $$z_{k,t}>0\;\mathrm{and}\;z_{k,t+1}>0$$

An emergent strategy satisfied:

(47) $$z_{k,t}=0\;\mathrm{and}{\;z}_{k,t+1}>0$$

A disappearing strategy satisfied:

(48) $$z_{k,t}>0\;\mathrm{and}\;z_{k,t+1}=0$$

A strategy absent from both states satisfied:

(49) $$z_{k,t}=0\;\mathrm{and}{\;z}_{k,t+1}=0$$

Because chromatographic zeros may indicate true absence or abundance below the detection threshold, emergence and disappearance describe analytical states rather than definitive biosynthetic activation or extinction.

### 3.13. Payoff Impact

A strategy can present a high positive payoff while occupying only a very small fraction of the chemical mixture. To distinguish relative directional advantage from quantitative influence, payoff impact was introduced as:

(50) $$I_{k,t}=g_{k,t}{\bar{z}}_{k,t}$$

where the mean abundance of the strategy during the transition was:

(51) $${\bar{z}}_{k,t}=\frac{z_{k,t}+z_{k,t+1}}{2}$$

Substituting Equation (51) into Equation (50):

(52) $$I_{k,t}=g_{k,t}\left(\frac{z_{k,t}+z_{k,t+1}}{2}\right)$$

The original normalized abundances, before zero replacement, were used in this equation.

The following interpretations were adopted:

$$g_{k,t}>0\;\mathrm{and}\;I_{k,t}\gg 0$$

indicate a major expanding chemical strategy;

$$g_{k,t}>0\;\mathrm{and}\;I_{k,t}\approx 0$$

indicate a rare but relatively expanding strategy;

$$g_{k,t}<0\;\mathrm{and}\;I_{k,t}\ll 0$$

indicate a quantitatively important contracting strategy;

and:

$$g_{k,t}<0\;\mathrm{and}\;I_{k,t}\approx 0$$

indicate a minor decline strategy.

Payoff impact is a supporting descriptor introduced in this article. It is not a previously established ecological or game-theoretical index.

### 3.14. Positive-Payoff Frequency and Temporal Consistency

For each strategy k and each ontogenetic transition, positive-payoff frequency was calculated as:

(53) $$F_{k}^{+}=\frac{1}{B}\sum_{b=1}^{B}1\left(g_{k,b}>0\right)$$

where B=5 is the number of monthly blocks (September to January).

Therefore:

(54) $$0\leq F_{k}^{+}\leq 1$$

A value of $F_{k}^{+}=1$ indicates positive payoff in all five monthly blocks.

A value of $F_{k}^{+}=0$ indicates that no monthly block presented a positive payoff.

Positive-payoff frequency measures directional consistency but is not equivalent to a statistical significance test.

For each strategy and transition, the following descriptive measures were calculated across the five monthly blocks: mean realized payoff; standard deviation; median realized payoff; mean payoff impact; positive-payoff frequency; number of evaluated blocks. 

The mean payoff was:

(55) $${\bar{g}}_{k}=\frac{1}{B}\sum_{b=1}^{B}g_{k,b}$$

The payoff standard deviation was:

(56) $$s_{g,k}=\sqrt{\frac{\sum_{b=1}^{B}{\left(g_{k,b}-{\bar{g}}_{k}\right)}^{2}}{B-1}}$$

The mean payoff impact was:

(57) $${\bar{I}}_{k}=\frac{1}{B}\sum_{b=1}^{B}I_{k,b}$$

### 3.15. Sensitivity of Payoff Direction to Zero Replacement

For every analytical hierarchy, month, transition, and strategy, the payoff obtained with the principal replacement value was denoted $g_{k,t}^{\left(0.5\right)}$.

The payoff obtained with the alternative replacement value was denoted $g_{k,t}^{\left(0.1\right)}$.

Directional agreement for an individual payoff was defined as:

(58) $$A_{k,t}=1\left[sgn\left(g_{k,t}^{\left(0.5\right)}\right)=sgn\left(g_{k,t}^{\left(0.1\right)}\right)\right]$$

The global directional agreement proportion was:

(59) $$A=\frac{1}{N_{g}}\sum_{k,t}A_{k,t}$$

where $N_{g}$ is the total number of paired payoff estimates.

The result satisfies:

(60) 0≤A≤1

A value approaching 1 indicates that payoff direction was largely unaffected by the magnitude of the zero-replacement value within the evaluated range.

This procedure evaluates robustness to the two specified replacement rules. It does not demonstrate robustness to every possible zero-imputation procedure.

### 3.16. Statistical Analysis

Monthly samples were treated as paired temporal blocks because each month contained leaf profiles and the four reproductive stages. Descriptive statistics were calculated across the five monthly blocks and reported as mean ± standard deviation.

The following state-level descriptors were included in inferential comparisons: Shannon diversity; Pielou evenness; GBDI; pathway entropy; abundance of the dominant pathway; abundance of the dominant metabolite. 

For descriptor Y, the blocked data structure was:

$$Y_{b,c}$$

where:

b=1, 2,…, 5

represents the monthly block, and:

c=1, 2,…, 5

represents leaves or one of the four reproductive stages.

Because only five temporal blocks were available and normality could not be reliably established, differences among compartments were evaluated using the nonparametric Friedman test [37] (Table S6).

For each descriptor, the null hypothesis was:

(61) $$H_{0}:\theta_{1}=\theta_{2}=\theta_{3}=\theta_{4}=\theta_{5}$$

where $\theta_{c}$ represents the central location of descriptor Y in compartment c.

The alternative hypothesis was:

(62) $$H_{1}:\theta_{c}\neq \theta_{c^{\prime }}\;\mathrm{for}\;\mathrm{at}\;\mathrm{least}\;\mathrm{one}\;\mathrm{pair}\;c\neq c^{\prime }$$

Within each block, ranks were assigned to the five compartments. Let $R_{c}$ represent the sum of the ranks assigned to compartment c across the B blocks. The Friedman statistic was:

(63) $$Q=\frac{12}{BK(K+1)}\sum_{c=1}^{K}R_{c}^{\;2}-3B(K+1)$$

where: B=5

and: K=5

The number of degrees of freedom was:

(64) df=K−1=4

When appropriate, the Friedman statistic was evaluated against the chi-squared distribution with four degrees of freedom.

Exploratory pairwise comparisons were performed using two-sided paired Wilcoxon signed-rank tests [38,39] (Table S7). For each descriptor, the number of possible pairwise comparisons was:

(65) M=52=5!2!3!=10

The raw p-values were ordered as $p_{\left(1\right)}\leq p_{\left(2\right)}\leq \cdots \leq p_{\left(M\right)}$.

Holm-adjusted values were calculated sequentially according to [39]:

(66) $$p_{Holm,(i)}=\underset{1\leq j\leq i}{max}\left[min\left(1,(M-j+1)p_{\left(j\right)}\right)\right]$$

The nominal statistical significance level was:

(67) $$\alpha_{stat}=0.05$$

The symbol $\alpha_{stat}$ was used here to distinguish the statistical significance threshold from the abundance-sensitivity exponent α=0.5 used in the GBDI calculation.

Because only five transition-specific payoff values were available for each strategy, formal hypothesis tests were not applied to individual metabolite, class, or pathway payoffs. These quantities were summarized using means, standard deviations, medians, impacts, and positive-payoff frequencies.

### 3.17. Computational Implementation and Reproducibility

All calculations were performed in Python 3.12.13 (Python Software Foundation, Wilmington, DE, USA) [40], using the open-source scientific packages NumPy 2.3.5 [41], Pandas 2.2.3 [42], and SciPy 1.17.0 [43]. Graphical representations were generated using Statistica software 12 (StatSoft Inc., Tulsa, OK, USA) and Matplotlib 3.10.8 (Matplotlib Development Team, Austin, TX, USA) [44]. Figures and schemes were exported as PNG files at 600 dpi.

**Figure 8:**
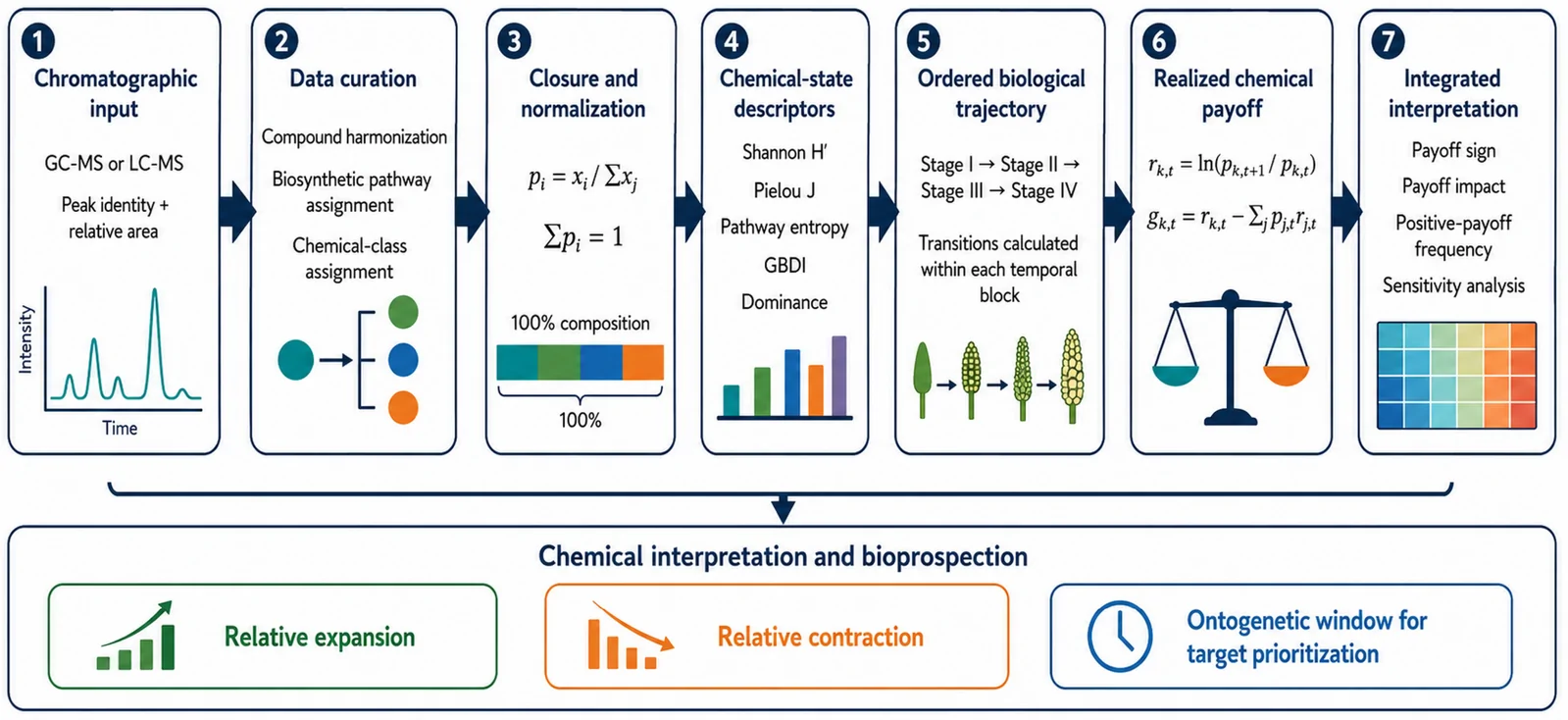
General analytical workflow for applying Chemical Game Theory to chromatographic chemodiversity data. GC–MS or LC–MS peak identity and abundance data are curated, harmonized, classified by chemical class and biosynthetic pathway, and normalized by compositional closure. Conventional chemical-state descriptors, including Shannon diversity, Pielou evenness, pathway entropy, dominance, and GBDI, are calculated before ordering samples along a biologically meaningful trajectory. Transition-specific log growth is centered relative to the abundance-weighted chemical background to obtain realized chemical payoff. Payoff sign, payoff impact, positive-payoff frequency, and zero-replacement sensitivity are subsequently integrated to identify relative chemical expansion, contraction, and candidate ontogenetic windows for target prioritization.

The workflow generated the following auditable outputs: (1) normalized compound-composition matrix; (2) compound-to-pathway classification; (3) compound-to-chemical-class classification; (4) state-level chemodiversity descriptors; (5) pathway-abundance profiles; (6) chemical-class profiles; (7) metabolite-level realized payoffs; (8) chemical-class-level realized payoffs; (9) biosynthetic-pathway-level realized payoffs; (10) payoff-impact summaries; (11) zero-replacement sensitivity analysis; (12) Friedman test results; (13) Wilcoxon–Holm pairwise comparisons (Figure 8).

To address computational reproducibility, the corresponding Python source code is now provided as Supplementary File S3. The archive contains the core calculation module, an executable analysis workflow, a validation script, the environment requirements, and a README file. The scripts implement compositional closure, state-level chemodiversity descriptors, GBDI, hierarchical realized-payoff calculations, payoff impact, zero-replacement sensitivity analysis, Shannon decomposition, and the Friedman and paired Wilcoxon–Holm procedures described above. Supplementary File S1 remains the canonical formula-driven data and numerical audit trail, whereas Supplementary File S3 provides the corresponding source-code implementation. The scripts are supplied for reproducibility and do not constitute a maintained software package, application programming interface, or standalone application.

## 4. Conclusions

Chemical Game Theory is presented as an operational analogy inspired by replicator dynamics for retrospective analysis of relative chromatographic profiles. The realized chemical payoff is a centered log-ratio descriptor of proportional enrichment or depletion, not a conventional payoff inferred from a game matrix and not a measure of Darwinian fitness or absolute biosynthetic production.

Application to *P. mollicomum* demonstrated that the proposed descriptors can separate compound-level diversity, annotation-dependent pathway organization, centered relative change, and abundance-weighted impact within one essential-oil dataset. Leaves had the highest numerical Shannon diversity and GBDI, but the Shannon difference was not significant. In the five observed blocks, the terpenoid and mixed categories showed opposite relative directions during the Stage I to II and Stage III to IV transitions. These results are descriptive and exploratory.

The distinction between realized payoff and payoff impact was essential. Payoff identified relative chemical expansion, including the emergence of rare strategies, whereas payoff impact identified changes with substantial quantitative influence on the entire mixture. Their combined use may support rational bioprospection by distinguishing developmental stages that maximize target abundance from stages representing active windows of relative chemical enrichment.

Chemical Game Theory does not attribute agency or Darwinian fitness to metabolites. The current data demonstrate the mathematical implementation, hierarchy dependence, sensitivity to zero replacement, and descriptive application of the framework in one GC–MS dataset of specialized volatile metabolites. Primary metabolism, nutrient status, disease state, and central metabolic flux may influence the observed specialized-metabolite phenotype, but they were not measured and are not inferred by this analysis. Proposed uses across chemically distinct essential oil datasets, broader LC–MS metabolomes, agricultural optimization, mechanistic biosynthetic interpretation, target prioritization, isolation success, and bioactivity-guided discovery remain hypotheses. They require independent biological replication, analytically appropriate coverage of the metabolite domain of interest, internal standards or response-factor correction when quantitative comparison is intended, absolute quantification, uncertainty-aware annotation, and prospective experimental validation.

## Figures and Tables

**Table 1:** Chemodiversity, biosynthetic architecture, and chemical dominance in leaves and reproductive stages of *Piper mollicomum*.

Compartment or Stage	Shannon H′	GBDI	Pielou *J*	Pathway Entropy Hpath	Dominant-Metabolite Abundance *p*max	Dominant-Pathway Abundance *P*max
Leaves	2.751 ± 0.322	1.735 ± 0.108	0.748	0.125	0.280	0.977
Stage I	1.787 ± 0.408	1.175 ± 0.195	0.701	0.626	0.388	0.668
Stage II	1.963 ± 0.640	1.265 ± 0.283	0.764	0.499	0.318	0.802
Stage III	2.214 ± 0.435	1.414 ± 0.163	0.671	0.554	0.356	0.763
Stage IV	1.925 ± 0.421	1.324 ± 0.156	0.668	0.425	0.407	0.848
Friedman *p*	0.1933	**0.0299**	0.0755	**0.0018**	0.2052	**0.0018**

Values are means ± standard deviations calculated from five monthly blocks. Pielou evenness and dominance variables are presented as monthly means. $p_{max}$ is the abundance of the dominant metabolite, and $P_{max}$ is the abundance of the dominant biosynthetic pathway. Friedman tests compared leaves and the four reproductive stages using month as the temporal block. **Bold** values indicate *p* < 0.05. Leaves were treated as a vegetative reference and not as an ontogenetic Stage 0.

**Table 2:** Mean relative allocation among the principal biosynthetic pathways in leaves and reproductive stages of *Piper mollicomum*.

Compartment or Stage	Terpenoid Pathway (%)	Mixed Pathway (%)	Shikimate Pathway (%)	Other Pathways (%)
Leaves	97.68 ± 1.37	0.52 ± 0.86	1.03 ± 0.76	0.77
Stage I	66.83 ± 9.31	33.07 ± 9.20	0.05 ± 0.11	0.05
Stage II	80.15 ± 7.81	19.40 ± 7.98	0.38 ± 0.60	0.07
Stage III	76.32 ± 10.54	23.00 ± 9.57	0.46 ± 0.72	0.22
Stage IV	84.78 ± 4.50	15.14 ± 4.46	0.08 ± 0.12	<0.01

Values are means ± standard deviations across the five monthly blocks. Eupatoriochromene was assigned to a mixed biosynthetic pathway, and benzyl benzoate was assigned to the shikimate pathway. Other pathways comprised fatty-acid/aliphatic and amino-acid-derived metabolites. Percentages were calculated from normalized individual chemical profiles.

**Table 3:** Realized payoffs of the principal biosynthetic pathways during reproductive development.

Transition	Biosynthetic Pathway	Mean Realized Payoff g	Positive Monthly Blocks	Mean Payoff Impact	Chemical Interpretation
Stage I → Stage II	Terpenoid	0.269 ± 0.191	5/5	0.196	Observed positive direction in 5/5 blocks
Stage I → Stage II	Mixed	−0.518 ± 0.418	0/5	−0.126	Observed negative direction in 5/5 blocks
Stage I → Stage II	Shikimate	0.837 ± 0.824	5/5	0.0037	High proportional change, low impact, zero-sensitive magnitude
Stage II → Stage III	Terpenoid	−0.002 ± 0.146	3/5	0.0018	Variable across blocks
Stage II → Stage III	Mixed	0.246 ± 0.802	2/5	0.0517	Variable across blocks
Stage II → Stage III	Shikimate	0.076 ± 0.696	4/5	0.0014	Small, variable component
Stage III → Stage IV	Terpenoid	0.153 ± 0.176	5/5	0.116	Observed positive direction in 5/5 blocks
Stage III → Stage IV	Mixed	−0.340 ± 0.204	0/5	−0.071	Observed negative direction in 5/5 blocks
Stage III → Stage IV	Shikimate	−0.594 ± 1.431	3/5	−0.0053	Low-abundance, unstable response

Values are means ± standard deviations across five monthly blocks. A positive payoff indicates expansion relative to the abundance-weighted chemical background, whereas a negative payoff indicates relative contraction. Payoff impact was calculated as the realized payoff multiplied by the mean initial–final abundance of the corresponding pathway. Positive monthly blocks indicate the number of months with g > 0.

**Table 4:** Relationship between realized payoff, impact and proposed chemical interpretation.

Realized Payoff	Abundance or Impact	Chemical Interpretation
Relatively large positive	Relatively large	Candidate major relative enrichment
Relatively large positive	Relatively small	Candidate minor relative enrichment, inspect zero sensitivity
Negative	Relatively large	Quantitatively influential relative depletion
Negative	Relatively small	Minor relative depletion

**Table 5:** Distribution and candidate ontogenetic windows of selected constituents.

Compound	Leaves (%)	Stage I (%)	Stage II (%)	Stage III (%)	Stage IV (%)	Highest Observed State
Linalool	14.19	10.12	19.54	20.59	28.34	Stage IV, September, 56.58%
1,8-Cineole	15.64	14.74	15.03	12.57	17.43	Stage IV, December, 37.43%
Eupatoriochromene	0.52	33.07	19.40	23.00	15.14	Stage I, November, 47.86%
α-Pinene	9.54	1.75	4.46	2.41	3.72	Leaves
β-Pinene	8.37	3.63	3.75	4.06	5.61	Leaves
Limonene	1.51	11.65	9.41	4.39	2.59	Stage I, September, 41.83%
Camphor	1.34	0.41	0.02	2.41	2.61	Stage IV, October, 12.70%
*E*-Caryophyllene	1.51	3.02	3.28	2.93	2.53	Stage II, December, 7.30%

Percentages are means of normalized profiles across the five monthly blocks. The final column reports the highest individual monthly value when relevant. These are isolated observed maxima, not statistically demonstrated target-yield windows. Across the eight compounds summarized in Table 5, no Friedman comparison among reproductive stages remained significant after Holm correction. The states are therefore candidate sampling states requiring independent replication and absolute yield measurements.

## Data Availability

This study did not generate new experimental data; it reanalyzed a previously published dataset from Piper mollicomum [31]. The complete compositional dataset used in the present analysis, computational workbook, statistical outputs, methodological notes, and Python source code are available in Supplementary Files S1–S3. The original data are reported in Supplementary Table S3 of the previously published study [31].

## Supplementary Materials

The following supporting information can be downloaded at: https://www.mdpi.com/article/10.3390/molecules31183325/s1, Supplementary File S1: complete GC–MS compositional dataset and auditable computational workbook, including original reported abundances, formula-driven compositional closure, normalized constituent proportions, biosynthetic-pathway and chemical-class assignments, state-level chemodiversity descriptors, Shannon diversity, GBDI, pathway and chemical-class profiles, complete constituent-, class-, and pathway-level logarithmic changes and realized relative payoffs, payoff impacts, zero-replacement sensitivity analysis, Friedman tests, paired Wilcoxon–Holm comparisons, variable definitions, and numerical validation outputs for leaves and four reproductive developmental stages of *Piper mollicomum*. Supplementary File S2: supplementary methodological notes, complete constituent classification, individual monthly chemodiversity and biosynthetic-architecture descriptors, monthly pathway-level realized payoffs, zero-replacement sensitivity results, complete statistical outputs, and Supplementary Figures S1–S3 [48,49]. Supplementary File S3: Python source-code archive for computational reproducibility, containing cgt_core.py, run_cgt_analysis.py, validate_cgt.py, requirements.txt, README.txt, and a code-availability note; the scripts implement compositional closure, chemodiversity descriptors, GBDI, hierarchical realized payoffs, payoff impact, zero-replacement sensitivity analyses, Shannon decomposition, and the reported nonparametric statistical procedures.

## Abbreviations

The following abbreviations are used in this manuscript:

A(k,t)	Original reported abundance of strategy k in state t
p(k,t)	Closed relative abundance of strategy k in state t
S	Chemical richness
*H′*	Shannon diversity
*J*	Pielou evenness
GBDI	General Biosynthetic Diversity Index
P(k,t)	Total abundance assigned to biosynthetic pathway k
α	GBDI abundance-sensitivity exponent
r(k,t)	Realized logarithmic change between consecutive states
$\bar{r}$(t)	Abundance-weighted mean logarithmic change
g(k,t)	Realized relative chemical payoff
I(k,t)	Abundance-weighted payoff impact
GC-MS	Gas chromatography–mass spectrometry
GC-FID	Gas chromatography–flame ionization detection
LC-MS	Liquid chromatography–mass spectrometry
LC-MS/MS	Liquid chromatography–tandem mass spectrometry
RI	Retention index
SD	Standard deviation
