# Supplementary material for: Chemical Game Theory: Linking Metabolite Diversity, Biosynthetic Architecture, and Realized Payoffs
Source: Molecules. 2026 Sep 19;31(18):3325. doi: 10.3390/molecules31183325 (PMC13609421; doi:10.3390/molecules31183325)
Supplement: Supplementary file 1 [file molecules-31-03325-s001.zip › Supplementary_File_S2_Chemical_Game_Theory_Extended_Tables_and_Figures.zip!/Supplementary_File_S2_Chemical_Game_Theory_Extended_Tables_and_Figures.pdf]

SUPPORTING INFORMATION

Chemical Game Theory: Linking Metabolite Diversity, Biosynthetic Architecture, and Realized Payoffs

Davyson de Lima Moreira, Renato Crespo Pereira, and Ygor Jessé Ramos

This document contains extended tables and figures supporting the proof-of-concept application of Chemical Game Theory to essential-oil profiles from leaves and four reproductive developmental stages of Piper mollicomum.

Contents of the Supporting Information

Supplementary Note S1: Dataset provenance, sample structure, and compositional closure
Supplementary Note S2: Analytical hierarchy, zero replacement, and interpretive limits
Table S1: Sample inventory and compositional-closure quality control
Table S2: Complete biosynthetic-pathway and chemical-class assignments
Table S3: State-level chemodiversity descriptors for every monthly block
Table S4: Monthly realized payoffs of the principal biosynthetic pathways
Table S5: Zero-replacement sensitivity summary
Table S6: Complete Friedman test results
Table S7: Complete paired Wilcoxon–Holm results
Figure S1: Monthly biosynthetic-pathway allocation
Figure S2: Sensitivity of payoff direction to zero replacement
Figure S3: Observed status of detected metabolite strategies

Supplementary Note S1. Dataset provenance and sample structure

The empirical matrix was reconstructed from the complete individual essential-oil profiles reported as “% in 1” in Supplementary Table S3 of the original Piper mollicomum study [S1]. The dataset contains five monthly blocks (September 2020–January 2021), each comprising leaves and reproductive Stages I–IV. Leaves were retained as a vegetative reference and were not treated as an ontogenetic Stage 0. Realized payoffs were calculated only for Stage I→Stage II, Stage II→Stage III, and Stage III→Stage IV within the same monthly block.

Each reported profile was normalized by compositional closure, $p_{k,t} = A_{k,t}/\Sigma_j A_{j,t}$. The closure step corrected differences between the total identified abundance and 100% but did not convert relative profiles into absolute metabolite concentrations. Consequently, all realized payoffs describe redistribution within the identified chemical mixture.

Supplementary Note S2. Analytical hierarchy and zero replacement

Chemical Game Theory was evaluated at three nested analytical levels: individual metabolites, chemical structural classes, and biosynthetic pathways. Eupatoriochromene was assigned to a mixed biosynthetic origin, whereas benzyl benzoate was assigned to the shikimate-derived pathway. The complete assignment table is provided as Table S2 and in Supplementary File S1.

Zeros were replaced before calculating logarithmic state-to-state changes. The principal analysis used one-half of the smallest positive abundance at the corresponding analytical hierarchy; the sensitivity analysis used one-tenth of that value. Replacement was followed by reclosure. Appearance and disappearance therefore describe analytical detection states and should not be interpreted as definitive biosynthetic activation or extinction.

Abbreviations

Abbreviation or symbol	Definition
$A_{k,t}$	Original chromatographic abundance of strategy k in state t
$p_{k,t}$	Closed relative abundance of strategy k in state t
S	Chemical richness
H′	Shannon diversity
J	Pielou evenness
GBDI	General Biosynthetic Diversity Index
Hpath	Between-pathway Shannon diversity
Pmax	Dominant-pathway abundance
pmax	Dominant-metabolite abundance
$r_{k,t}$	Realized logarithmic change
$\bar{r}_t$	Abundance-weighted mean logarithmic change
$g_{k,t}$	Realized relative chemical payoff
GC–MS	Gas chromatography–mass spectrometry
GC–FID	Gas chromatography–flame ionization detection
LC–MS	Liquid chromatography–mass spectrometry
RI	Retention index
SD	Standard deviation

Table S1. Sample inventory and compositional-closure quality control for all 25 chemical states.

Month	State	Reported rows	Positive constituents	Reported total (%)	Closed total	Minimum positive p	Maximum p
Sep 2020	Leaves	53	53	93.17	1.000000	0.001073	0.398841
Sep 2020	Stage I	6	6	99.96	1.000000	0.054522	0.418267
Sep 2020	Stage II	6	6	99.97	1.000000	0.029409	0.353006
Sep 2020	Stage III	12	12	84.31	1.000000	0.000830	0.558415
Sep 2020	Stage IV	27	27	95.62	1.000000	0.000523	0.565781
Oct 2020	Leaves	45	45	93.14	1.000000	0.001718	0.239747
Oct 2020	Stage I	9	9	85.07	1.000000	0.008111	0.415187
Oct 2020	Stage II	5	5	84.06	1.000000	0.028789	0.434571
Oct 2020	Stage III	35	35	97.14	1.000000	0.001235	0.311612
Oct 2020	Stage IV	7	7	98.18	1.000000	0.021186	0.555612
Nov 2020	Leaves	41	41	94.48	1.000000	0.001164	0.156118
Nov 2020	Stage I	12	12	69.71	1.000000	0.002008	0.478554
Nov 2020	Stage II	28	28	85.20	1.000000	0.001174	0.278638
Nov 2020	Stage III	35	35	88.85	1.000000	0.001238	0.272932
Nov 2020	Stage IV	30	30	92.55	1.000000	0.001837	0.216532
Dec 2020	Leaves	27	27	86.20	1.000000	0.002088	0.299304
Dec 2020	Stage I	33	33	80.76	1.000000	0.001857	0.347449
Dec 2020	Stage II	37	37	86.33	1.000000	0.001738	0.164022
Dec 2020	Stage III	30	30	64.73	1.000000	0.002008	0.364591
Dec 2020	Stage IV	16	16	92.43	1.000000	0.001731	0.374337
Jan 2021	Leaves	36	36	92.15	1.000000	0.002170	0.305914
Jan 2021	Stage I	19	19	70.10	1.000000	0.001854	0.282596
Jan 2021	Stage II	15	15	58.16	1.000000	0.002579	0.361761
Jan 2021	Stage III	29	29	87.66	1.000000	0.001255	0.273557
Jan 2021	Stage IV	23	23	56.16	1.000000	0.002315	0.322472

Note: The normalized total equals 1.000000 for every chemical state. Reported rows include zero-valued records where present in the source table.

Table S2. Complete auditable assignment of essential-oil constituents to biosynthetic pathways and chemical classes.

Reported compound	Canonical compound	Biosynthetic pathway	Chemical class
2,3-diethyl-pyrazine	2,3-diethyl-pyrazine	Amino-acid-derived	Nitrogenous derivative
(Z)-3-Hexenol	(z)-3-hexenol	Fatty-acid/aliphatic	Aliphatic derivative
2E,4E-dodecadienal	2e,4e-dodecadienal	Fatty-acid/aliphatic	Aliphatic derivative
3E-Hexenol	3e-hexenol	Fatty-acid/aliphatic	Aliphatic derivative
tridecanone	tridecanone	Fatty-acid/aliphatic	Aliphatic derivative
Tridecanone	tridecanone	Fatty-acid/aliphatic	Aliphatic derivative
undecanol	undecanol	Fatty-acid/aliphatic	Aliphatic derivative
undecanone	undecanone	Fatty-acid/aliphatic	Aliphatic derivative
Eupatoriochromene	eupatoriochromene	Mixed	Mixed chromene
eupatoriochromene	eupatoriochromene	Mixed	Mixed chromene
benzyl benzoate	benzyl benzoate	Shikimate	Shikimate benzenoid
tolualdehyde	tolualdehyde	Shikimate	Shikimate benzenoid
allo-ocimene	allo-ocimene	Terpenoid	Monoterpene hydrocarbon
camphene	camphene	Terpenoid	Monoterpene hydrocarbon
E-β-ocimene	e-β-ocimene	Terpenoid	Monoterpene hydrocarbon
limonene	limonene	Terpenoid	Monoterpene hydrocarbon
Myrcene	myrcene	Terpenoid	Monoterpene hydrocarbon
myrcene	myrcene	Terpenoid	Monoterpene hydrocarbon
Ocimene	ocimene	Terpenoid	Monoterpene hydrocarbon
ocimene	ocimene	Terpenoid	Monoterpene hydrocarbon
sabinene	sabinene	Terpenoid	Monoterpene hydrocarbon
Sabinene	sabinene	Terpenoid	Monoterpene hydrocarbon
Terpinolene	terpinolene	Terpenoid	Monoterpene hydrocarbon
terpinolene	terpinolene	Terpenoid	Monoterpene hydrocarbon
Z-β-ocimene	z-β-ocimene	Terpenoid	Monoterpene hydrocarbon
α-phellandrene	α-phellandrene	Terpenoid	Monoterpene hydrocarbon
α-pinene	α-pinene	Terpenoid	Monoterpene hydrocarbon
α-terpinene	α-terpinene	Terpenoid	Monoterpene hydrocarbon
β-pinene	β-pinene	Terpenoid	Monoterpene hydrocarbon
γ-terpinene	γ-terpinene	Terpenoid	Monoterpene hydrocarbon
sclareolide	sclareolide	Terpenoid	Oxygenated diterpene
1,8-cineole	1,8-cineole	Terpenoid	Oxygenated monoterpene
borneol	borneol	Terpenoid	Oxygenated monoterpene
bornyl acetate	bornyl acetate	Terpenoid	Oxygenated monoterpene
camphene hydrate	camphene hydrate	Terpenoid	Oxygenated monoterpene
camphor	camphor	Terpenoid	Oxygenated monoterpene
cis-linalool oxide	cis-linalool oxide	Terpenoid	Oxygenated monoterpene
Geraniol	geraniol	Terpenoid	Oxygenated monoterpene
isopulegyl acetate	isopulegyl acetate	Terpenoid	Oxygenated monoterpene
linalool	linalool	Terpenoid	Oxygenated monoterpene
Linalool	linalool	Terpenoid	Oxygenated monoterpene
linalool isovalerate	linalool isovalerate	Terpenoid	Oxygenated monoterpene
linalool oxide <cis->	linalool oxide <cis->	Terpenoid	Oxygenated monoterpene
linalool propanoate	linalool propanoate	Terpenoid	Oxygenated monoterpene
neryl acetate	neryl acetate	Terpenoid	Oxygenated monoterpene
pinocamphone	pinocamphone	Terpenoid	Oxygenated monoterpene
terpinen-4-ol	terpinen-4-ol	Terpenoid	Oxygenated monoterpene
trans-sabinene hydrate	trans-sabinene hydrate	Terpenoid	Oxygenated monoterpene
trans-β-terpineol	trans-β-terpineol	Terpenoid	Oxygenated monoterpene
α-fenchocamphorone	α-fenchocamphorone	Terpenoid	Oxygenated monoterpene
α-terpineol	α-terpineol	Terpenoid	Oxygenated monoterpene
δ-terpineol	δ-terpineol	Terpenoid	Oxygenated monoterpene
10-epi-γ-eudesmol	10-epi-γ-eudesmol	Terpenoid	Oxygenated sesquiterpene
aromadendrene epoxide	aromadendrene epoxide	Terpenoid	Oxygenated sesquiterpene
caryophyllene acetate	caryophyllene acetate	Terpenoid	Oxygenated sesquiterpene
caryophyllene oxide	caryophyllene oxide	Terpenoid	Oxygenated sesquiterpene
cis-thujopsenal	cis-thujopsenal	Terpenoid	Oxygenated sesquiterpene
E-nerolidol	e-nerolidol	Terpenoid	Oxygenated sesquiterpene
elemenone	elemenone	Terpenoid	Oxygenated sesquiterpene
Elemol	elemol	Terpenoid	Oxygenated sesquiterpene
elemol acetate	elemol acetate	Terpenoid	Oxygenated sesquiterpene
epi-α-cadinol	epi-α-cadinol	Terpenoid	Oxygenated sesquiterpene
eudesm-7(11)-en-4-ol	eudesm-7(11)-en-4-ol	Terpenoid	Oxygenated sesquiterpene
germacrene D-4-ol	germacrene d-4-ol	Terpenoid	Oxygenated sesquiterpene
gleenol	gleenol	Terpenoid	Oxygenated sesquiterpene
guaiol	guaiol	Terpenoid	Oxygenated sesquiterpene
intermedeol	intermedeol	Terpenoid	Oxygenated sesquiterpene
Ledol	ledol	Terpenoid	Oxygenated sesquiterpene
maaliol	maaliol	Terpenoid	Oxygenated sesquiterpene
pogostol	pogostol	Terpenoid	Oxygenated sesquiterpene
Santalol acetate <(Z)-α->	santalol acetate <(z)-α->	Terpenoid	Oxygenated sesquiterpene
selin-11-en-4-α-ol	selin-11-en-4-α-ol	Terpenoid	Oxygenated sesquiterpene
spathulenol	spathulenol	Terpenoid	Oxygenated sesquiterpene
Spathulenol	spathulenol	Terpenoid	Oxygenated sesquiterpene
Viridiflorol	viridiflorol	Terpenoid	Oxygenated sesquiterpene

Reported compound	Canonical compound	Biosynthetic pathway	Chemical class
Z-bisaboladien-4-ol	z-bisaboladien-4-ol	Terpenoid	Oxygenated sesquiterpene
zerumbone	zerumbone	Terpenoid	Oxygenated sesquiterpene
Zerumbone	zerumbone	Terpenoid	Oxygenated sesquiterpene
α-cadinol	α-cadinol	Terpenoid	Oxygenated sesquiterpene
α-himachal-4-en-1-β-ol	α-himachal-4-en-1-β-ol	Terpenoid	Oxygenated sesquiterpene
α-muurolol	α-muurolol	Terpenoid	Oxygenated sesquiterpene
9-epi-E-caryophyllene	9-epi-e-caryophyllene	Terpenoid	Sesquiterpene hydrocarbon
aromadendrene	aromadendrene	Terpenoid	Sesquiterpene hydrocarbon
bicyclogermacrene	bicyclogermacrene	Terpenoid	Sesquiterpene hydrocarbon
cis-4,10-epoxy-amorphane	cis-4,10-epoxy-amorphane	Terpenoid	Sesquiterpene hydrocarbon
cis-cadina-1(6),4-diene	cis-cadina-1(6),4-diene	Terpenoid	Sesquiterpene hydrocarbon
cis-cadina-1,4-diene	cis-cadina-1,4-diene	Terpenoid	Sesquiterpene hydrocarbon
cis-muurola-3,5-diene	cis-muurola-3,5-diene	Terpenoid	Sesquiterpene hydrocarbon
cis-muurola-4(14),5-diene	cis-muurola-4(14),5-diene	Terpenoid	Sesquiterpene hydrocarbon
cis-β-guaiene	cis-β-guaiene	Terpenoid	Sesquiterpene hydrocarbon
Cubebene <β->	cubebene <β->	Terpenoid	Sesquiterpene hydrocarbon
E,E-α-farnesene	e,e-α-farnesene	Terpenoid	Sesquiterpene hydrocarbon
E-caryophyllene	e-caryophyllene	Terpenoid	Sesquiterpene hydrocarbon
E-iso-γ-bisabolene	e-iso-γ-bisabolene	Terpenoid	Sesquiterpene hydrocarbon
epizonarene	epizonarene	Terpenoid	Sesquiterpene hydrocarbon
germacrene B	germacrene b	Terpenoid	Sesquiterpene hydrocarbon
germacrene D	germacrene d	Terpenoid	Sesquiterpene hydrocarbon
isoledene	isoledene	Terpenoid	Sesquiterpene hydrocarbon
trans-cadina-1(6),4-diene	trans-cadina-1(6),4-diene	Terpenoid	Sesquiterpene hydrocarbon
trans-muurola-4(14),5-diene	trans-muurola-4(14),5-diene	Terpenoid	Sesquiterpene hydrocarbon
valencene	valencene	Terpenoid	Sesquiterpene hydrocarbon
Z-α-bisabolene	z-α-bisabolene	Terpenoid	Sesquiterpene hydrocarbon
α-amorphene	α-amorphene	Terpenoid	Sesquiterpene hydrocarbon
α-cedrene	α-cedrene	Terpenoid	Sesquiterpene hydrocarbon
α-copaene	α-copaene	Terpenoid	Sesquiterpene hydrocarbon
α-cubebene	α-cubebene	Terpenoid	Sesquiterpene hydrocarbon
α-gurjunene	α-gurjunene	Terpenoid	Sesquiterpene hydrocarbon
α-humulene	α-humulene	Terpenoid	Sesquiterpene hydrocarbon
α-muurolene	α-muurolene	Terpenoid	Sesquiterpene hydrocarbon
α-selinene	α-selinene	Terpenoid	Sesquiterpene hydrocarbon
α-ylangene	α-ylangene	Terpenoid	Sesquiterpene hydrocarbon
β-bisabolene	β-bisabolene	Terpenoid	Sesquiterpene hydrocarbon
β-elemene	β-elemene	Terpenoid	Sesquiterpene hydrocarbon
β-gurjunene	β-gurjunene	Terpenoid	Sesquiterpene hydrocarbon
β-panasinsene	β-panasinsene	Terpenoid	Sesquiterpene hydrocarbon
β-selinene	β-selinene	Terpenoid	Sesquiterpene hydrocarbon
γ-cadinene	γ-cadinene	Terpenoid	Sesquiterpene hydrocarbon
γ-curcumene	γ-curcumene	Terpenoid	Sesquiterpene hydrocarbon
γ-elemene	γ-elemene	Terpenoid	Sesquiterpene hydrocarbon
γ-gurjunene	γ-gurjunene	Terpenoid	Sesquiterpene hydrocarbon
γ-himachalene	γ-himachalene	Terpenoid	Sesquiterpene hydrocarbon
δ-cadinene	δ-cadinene	Terpenoid	Sesquiterpene hydrocarbon
δ-elemene	δ-elemene	Terpenoid	Sesquiterpene hydrocarbon
δ-selinene	δ-selinene	Terpenoid	Sesquiterpene hydrocarbon

Note: Eupatoriochromene was assigned to mixed biosynthetic origin; benzyl benzoate was assigned to the shikimate-derived pathway. Assignments affect pathway-sensitive descriptors but not compound-level Shannon diversity.

Table S3. State-level chemodiversity and biosynthetic-architecture descriptors for every monthly block.

Month	State	S	Shannon H′	Simpson 1−D	Pielou J	Hill ^{1}D	Hill ^{2}D	pmax	Top 3	Pathway richness	Hpath	$^{1}D_{path}$	Pmax	GBDI
Sep 2020	Leaves	53	2.7170	0.8238	0.6843	15.1352	5.6747	0.3988	0.5213	3	0.1409	1.1514	0.9725	1.8034
Sep 2020	Stage I	6	1.5267	0.7351	0.8521	4.6031	3.7756	0.4183	0.7819	2	0.5469	1.7279	0.7636	1.0332
Sep 2020	Stage II	6	1.5272	0.7560	0.8524	4.6054	4.0989	0.3530	0.7843	2	0.5214	1.6843	0.7843	1.0323
Sep 2020	Stage III	12	1.4387	0.6425	0.5790	4.2152	2.7975	0.5584	0.8431	2	0.4081	1.5040	0.8583	1.1382
Sep 2020	Stage IV	27	1.6777	0.6516	0.5090	5.3534	2.8706	0.5658	0.7657	3	0.4004	1.4924	0.8690	1.3334
Oct 2020	Leaves	45	3.0300	0.9143	0.7960	20.6975	11.6649	0.2397	0.3616	3	0.1215	1.1292	0.9777	1.8232
Oct 2020	Stage I	9	1.4633	0.7031	0.6660	4.3202	3.3676	0.4152	0.8882	2	0.6180	1.8551	0.6914	1.0480
Oct 2020	Stage II	5	1.2885	0.6861	0.8006	3.6272	3.1862	0.4346	0.9291	2	0.5505	1.7341	0.7605	0.9485
Oct 2020	Stage III	35	2.4280	0.8523	0.6829	11.3362	6.7715	0.3116	0.5655	2	0.3976	1.4883	0.8640	1.5670
Oct 2020	Stage IV	7	1.4341	0.6517	0.7370	4.1961	2.8710	0.5556	0.7832	2	0.3262	1.3858	0.8995	1.1016
Nov 2020	Leaves	41	3.1006	0.9315	0.8349	22.2122	14.6003	0.1561	0.3816	4	0.2192	1.2451	0.9560	1.7905
Nov 2020	Stage I	12	1.5084	0.6895	0.6070	4.5197	3.2201	0.4786	0.8403	3	0.7085	2.0310	0.5189	1.0285
Nov 2020	Stage II	28	2.1350	0.8192	0.6407	8.4570	5.5314	0.2786	0.6831	3	0.6075	1.8357	0.7190	1.3750
Nov 2020	Stage III	35	2.3967	0.8423	0.6741	10.9873	6.3428	0.2729	0.6268	4	0.6444	1.9048	0.7173	1.4751
Nov 2020	Stage IV	30	2.5116	0.8814	0.7385	12.3250	8.4338	0.2165	0.5139	3	0.5354	1.7082	0.7816	1.5097
Dec 2020	Leaves	27	2.3195	0.8458	0.7038	10.1708	6.4838	0.2993	0.5971	3	0.0597	1.0615	0.9906	1.5636
Dec 2020	Stage I	33	2.3635	0.8303	0.6760	10.6284	5.8944	0.3474	0.6187	3	0.6614	1.9376	0.6502	1.4488
Dec 2020	Stage II	37	2.9440	0.9214	0.8153	18.9907	12.7210	0.1640	0.3971	4	0.5402	1.7164	0.8184	1.6551
Dec 2020	Stage III	30	2.4589	0.8320	0.7229	11.6918	5.9526	0.3646	0.5843	4	0.7742	2.1688	0.6112	1.4153
Dec 2020	Stage IV	16	1.8414	0.7839	0.6641	6.3052	4.6279	0.3743	0.7069	2	0.4599	1.5840	0.8274	1.2554
Jan 2021	Leaves	36	2.5883	0.8621	0.7223	13.3066	7.2519	0.3059	0.5557	4	0.0815	1.0849	0.9872	1.6945
Jan 2021	Stage I	19	2.0746	0.8145	0.7046	7.9614	5.3922	0.2826	0.6924	2	0.5954	1.8137	0.7174	1.3142
Jan 2021	Stage II	15	1.9199	0.7913	0.7089	6.8200	4.7905	0.3618	0.7118	3	0.2766	1.3187	0.9254	1.3163
Jan 2021	Stage III	29	2.3480	0.8449	0.6973	10.4647	6.4458	0.2736	0.6204	2	0.5450	1.7246	0.7652	1.4743
Jan 2021	Stage IV	23	2.1601	0.8299	0.6889	8.6721	5.8806	0.3225	0.6250	2	0.4023	1.4952	0.8615	1.4184

Note: The main article reports compartment-level means ± SD. This table provides the individual monthly values underlying those summaries.

Table S4. Monthly realized payoffs of the principal biosynthetic pathways during reproductive development.

Month	Transition	Pathway	ε	pinitial	pfinal	r	g	Payoff impact	Status
Sep 2020	Stage I→Stage II	Terpenoid	0.000918	0.763605	0.784335	0.026785	0.028024	0.021690	persistent
Sep 2020	Stage I→Stage II	Mixed	0.000918	0.236395	0.215665	-0.091777	-0.090539	-0.020464	persistent
Sep 2020	Stage I→Stage II	Shikimate	0.000918	0.000000	0.000000	-2.220e-16	0.001239	0.000000	absent
Oct 2020	Stage I→Stage II	Terpenoid	0.000918	0.691431	0.760528	0.095250	0.107583	0.078103	persistent
Oct 2020	Stage I→Stage II	Mixed	0.000918	0.308569	0.239472	-0.253511	-0.241178	-0.066088	persistent
Oct 2020	Stage I→Stage II	Shikimate	0.000918	0.000000	0.000000	0.000000	0.012333	0.000000	absent
Nov 2020	Stage I→Stage II	Terpenoid	0.000918	0.518864	0.719014	0.326239	0.417433	0.258366	persistent
Nov 2020	Stage I→Stage II	Mixed	0.000918	0.478554	0.278638	-0.540854	-0.449660	-0.170240	persistent
Nov 2020	Stage I→Stage II	Shikimate	0.000918	0.000000	0.002347	0.938414	1.029607	0.001208	emergence
Dec 2020	Stage I→Stage II	Terpenoid	0.000918	0.650198	0.818371	0.230956	0.335648	0.246461	persistent
Dec 2020	Stage I→Stage II	Mixed	0.000918	0.347449	0.164022	-0.749702	-0.645010	-0.164952	persistent
Dec 2020	Stage I→Stage II	Shikimate	0.000918	0.002353	0.014248	1.801967	1.906659	0.015826	persistent
Jan 2021	Stage I→Stage II	Terpenoid	0.000918	0.717404	0.925378	0.255480	0.456675	0.375109	persistent
Jan 2021	Stage I→Stage II	Mixed	0.000918	0.282596	0.072043	-1.365845	-1.164650	-0.206515	persistent
Jan 2021	Stage I→Stage II	Shikimate	0.000918	0.000000	0.002579	1.033452	1.234647	0.001592	emergence
Sep 2020	Stage II→Stage III	Terpenoid	0.000918	0.784335	0.858261	0.090072	0.109894	0.090255	persistent
Sep 2020	Stage II→Stage III	Mixed	0.000918	0.215665	0.141739	-0.419739	-0.399917	-0.071466	persistent
Sep 2020	Stage II→Stage III	Shikimate	0.000918	0.000000	0.000000	2.220e-16	0.019822	0.000000	absent
Oct 2020	Stage II→Stage III	Terpenoid	0.000918	0.760528	0.864011	0.127572	0.165952	0.134797	persistent
Oct 2020	Stage II→Stage III	Mixed	0.000918	0.239472	0.135989	-0.565860	-0.527480	-0.099024	persistent
Oct 2020	Stage II→Stage III	Shikimate	0.000918	0.000000	0.000000	2.220e-16	0.038380	0.000000	absent
Nov 2020	Stage II→Stage III	Terpenoid	0.000918	0.719014	0.717276	-0.001503	0.001523	0.001094	persistent
Nov 2020	Stage II→Stage III	Mixed	0.000918	0.278638	0.272932	-0.019776	-0.016750	-0.004619	persistent
Nov 2020	Stage II→Stage III	Shikimate	0.000918	0.002347	0.006415	1.006288	1.009314	0.004422	persistent
Dec 2020	Stage II→Stage III	Terpenoid	0.000918	0.818371	0.611154	-0.291967	-0.189055	-0.135130	persistent
Dec 2020	Stage II→Stage III	Mixed	0.000918	0.164022	0.364591	0.798778	0.901690	0.238323	persistent
Dec 2020	Stage II→Stage III	Shikimate	0.000918	0.014248	0.016530	0.148597	0.251509	0.003870	persistent
Jan 2021	Stage II→Stage III	Terpenoid	0.000918	0.925378	0.765229	-0.190943	-0.096796	-0.081822	persistent

Month	Transition	Pathway	ε	pinitial	pfinal	r	g	Payoff impact	Status
Jan 2021	Stage II→Stage III	Mixed	0.000918	0.072043	0.234771	1.180435	1.274582	0.195529	persistent
Jan 2021	Stage II→Stage III	Shikimate	0.000918	0.002579	0.000000	-1.033452	-0.939304	-0.001211	disappearance
Sep 2020	Stage III→Stage IV	Terpenoid	0.000918	0.858261	0.868960	0.013305	0.014616	0.012623	persistent
Sep 2020	Stage III→Stage IV	Mixed	0.000918	0.141739	0.128634	-0.096097	-0.094786	-0.012814	persistent
Sep 2020	Stage III→Stage IV	Shikimate	0.000918	0.000000	0.002405	0.963711	0.965022	0.001161	emergence
Oct 2020	Stage III→Stage IV	Terpenoid	0.000918	0.864011	0.899470	0.040221	0.046538	0.041034	persistent
Oct 2020	Stage III→Stage IV	Mixed	0.000918	0.135989	0.100530	-0.302124	-0.295807	-0.034982	persistent
Oct 2020	Stage III→Stage IV	Shikimate	0.000918	0.000000	0.000000	-2.220e-16	0.006317	0.000000	absent
Nov 2020	Stage III→Stage IV	Terpenoid	0.000918	0.717276	0.781632	0.085005	0.099878	0.074854	persistent
Nov 2020	Stage III→Stage IV	Mixed	0.000918	0.272932	0.216532	-0.232403	-0.217530	-0.053236	persistent
Nov 2020	Stage III→Stage IV	Shikimate	0.000918	0.006415	0.001837	-1.251555	-1.236681	-0.005103	persistent
Dec 2020	Stage III→Stage IV	Terpenoid	0.000918	0.611154	0.827437	0.301150	0.454621	0.327006	persistent
Dec 2020	Stage III→Stage IV	Mixed	0.000918	0.364591	0.172563	-0.749848	-0.596378	-0.160174	persistent
Dec 2020	Stage III→Stage IV	Shikimate	0.000918	0.016530	0.000000	-2.892120	-2.738650	-0.022635	disappearance
Jan 2021	Stage III→Stage IV	Terpenoid	0.000918	0.765229	0.861467	0.118462	0.151562	0.123273	persistent
Jan 2021	Stage III→Stage IV	Mixed	0.000918	0.234771	0.138533	-0.527502	-0.494402	-0.092281	persistent
Jan 2021	Stage III→Stage IV	Shikimate	0.000918	0.000000	0.000000	2.220e-16	0.033101	0.000000	absent

Note: ε is the principal zero-replacement value at pathway level. $g > 0$ indicates relative expansion and $g < 0$ relative contraction against the abundance-weighted chemical background.

Table S5. Sensitivity of realized-payoff direction to the zero-replacement factor.

Hierarchy	Transition	Paired payoffs	Same sign	Directional agreement (%)	Mean |Δg|	Maximum |Δg|
Biosynthetic pathway	Stage I→Stage II	25	25	100.00	0.258422	1.614120
Biosynthetic pathway	Stage II→Stage III	25	25	100.00	0.129443	1.610104
Biosynthetic pathway	Stage III→Stage IV	25	25	100.00	0.259830	1.609854
Chemical class	Stage I→Stage II	45	45	100.00	0.259598	1.617028
Chemical class	Stage II→Stage III	45	45	100.00	0.108888	1.612268
Chemical class	Stage III→Stage IV	45	45	100.00	0.195430	1.609906
Metabolite	Stage I→Stage II	450	448	99.56	0.366562	2.123891
Metabolite	Stage II→Stage III	345	344	99.71	0.384511	1.886038
Metabolite	Stage III→Stage IV	345	344	99.71	0.445571	2.023389

Note: Directional agreement compares payoff signs under replacement by one-half versus one-tenth of the smallest positive abundance. It does not test other imputation procedures.

Table S6. Complete Friedman test results for blocked comparisons among leaves and reproductive Stages I–IV.

Descriptor	Friedman χ^2	df	p-value
Shannon H′	6.0800	4	0.193253
Pielou J	8.4800	4	0.075496
GBDI	10.7200	4	0.029898
Pathway entropy Hpath	17.1200	4	0.001832
Dominant-pathway abundance Pmax	17.1200	4	0.001832
Dominant-metabolite abundance pmax	5.9200	4	0.205203

Note: Month was treated as the block (n = 5). The analyses are exploratory because of the limited number of temporal blocks.

Table S7. Complete two-sided paired Wilcoxon signed-rank comparisons with Holm adjustment.

Descriptor	Comparison	Wilcoxon W	Raw p-value	Holm-adjusted p-value
Shannon H′	Leaves vs Stage I	1.00	0.125000	1.000000
Shannon H′	Leaves vs Stage II	1.00	0.125000	1.000000
Shannon H′	Leaves vs Stage III	1.00	0.125000	1.000000
Shannon H′	Leaves vs Stage IV	0.00	0.062500	0.625000
Shannon H′	Stage I vs Stage II	5.00	0.625000	1.000000
Shannon H′	Stage I vs Stage III	1.00	0.125000	1.000000
Shannon H′	Stage I vs Stage IV	5.00	0.625000	1.000000
Shannon H′	Stage II vs Stage III	5.00	0.625000	1.000000
Shannon H′	Stage II vs Stage IV	5.00	0.625000	1.000000
Shannon H′	Stage III vs Stage IV	4.00	0.437500	1.000000
Pielou J	Leaves vs Stage I	4.00	0.437500	1.000000
Pielou J	Leaves vs Stage II	7.00	1.000000	1.000000
Pielou J	Leaves vs Stage III	1.00	0.125000	1.000000
Pielou J	Leaves vs Stage IV	0.00	0.062500	0.625000
Pielou J	Stage I vs Stage II	0.00	0.062500	0.625000
Pielou J	Stage I vs Stage III	6.00	0.812500	1.000000
Pielou J	Stage I vs Stage IV	7.00	1.000000	1.000000
Pielou J	Stage II vs Stage III	2.00	0.187500	1.000000
Pielou J	Stage II vs Stage IV	3.00	0.312500	1.000000
Pielou J	Stage III vs Stage IV	6.00	0.812500	1.000000
GBDI	Leaves vs Stage I	0.00	0.062500	0.625000
GBDI	Leaves vs Stage II	1.00	0.125000	0.875000
GBDI	Leaves vs Stage III	0.00	0.062500	0.625000
GBDI	Leaves vs Stage IV	0.00	0.062500	0.625000
GBDI	Stage I vs Stage II	4.00	0.437500	1.000000
GBDI	Stage I vs Stage III	1.00	0.125000	0.875000
GBDI	Stage I vs Stage IV	3.00	0.312500	1.000000
GBDI	Stage II vs Stage III	4.00	0.437500	1.000000
GBDI	Stage II vs Stage IV	5.00	0.625000	1.000000
GBDI	Stage III vs Stage IV	5.00	0.625000	1.000000
Pathway entropy Hpath	Leaves vs Stage I	0.00	0.062500	0.625000
Pathway entropy Hpath	Leaves vs Stage II	0.00	0.062500	0.625000
Pathway entropy Hpath	Leaves vs Stage III	0.00	0.062500	0.625000
Pathway entropy Hpath	Leaves vs Stage IV	0.00	0.062500	0.625000
Pathway entropy Hpath	Stage I vs Stage II	0.00	0.062500	0.625000
Pathway entropy Hpath	Stage I vs Stage III	3.00	0.312500	0.937500
Pathway entropy Hpath	Stage I vs Stage IV	0.00	0.062500	0.625000
Pathway entropy Hpath	Stage II vs Stage III	5.00	0.625000	0.937500
Pathway entropy Hpath	Stage II vs Stage IV	4.00	0.437500	0.937500
Pathway entropy Hpath	Stage III vs Stage IV	0.00	0.062500	0.625000
Dominant-pathway abundance Pmax	Leaves vs Stage I	0.00	0.062500	0.625000
Dominant-pathway abundance Pmax	Leaves vs Stage II	0.00	0.062500	0.625000
Dominant-pathway abundance Pmax	Leaves vs Stage III	0.00	0.062500	0.625000
Dominant-pathway abundance Pmax	Leaves vs Stage IV	0.00	0.062500	0.625000
Dominant-pathway abundance Pmax	Stage I vs Stage II	0.00	0.062500	0.625000
Dominant-pathway abundance Pmax	Stage I vs Stage III	1.00	0.125000	0.625000
Dominant-pathway abundance Pmax	Stage I vs Stage IV	0.00	0.062500	0.625000
Dominant-pathway abundance Pmax	Stage II vs Stage III	5.00	0.625000	0.625000
Dominant-pathway abundance Pmax	Stage II vs Stage IV	3.00	0.312500	0.625000
Dominant-pathway abundance Pmax	Stage III vs Stage IV	0.00	0.062500	0.625000

Descriptor	Comparison	Wilcoxon W	Raw p-value	Holm-adjusted p-value
Dominant-metabolite abundance pmax	Leaves vs Stage I	2.00	0.187500	1.000000
Dominant-metabolite abundance pmax	Leaves vs Stage II	5.00	0.625000	1.000000
Dominant-metabolite abundance pmax	Leaves vs Stage III	1.00	0.125000	1.000000
Dominant-metabolite abundance pmax	Leaves vs Stage IV	0.00	0.062500	0.625000
Dominant-metabolite abundance pmax	Stage I vs Stage II	4.00	0.437500	1.000000
Dominant-metabolite abundance pmax	Stage I vs Stage III	6.00	0.812500	1.000000
Dominant-metabolite abundance pmax	Stage I vs Stage IV	5.00	0.625000	1.000000
Dominant-metabolite abundance pmax	Stage II vs Stage III	6.00	0.812500	1.000000
Dominant-metabolite abundance pmax	Stage II vs Stage IV	3.00	0.312500	1.000000
Dominant-metabolite abundance pmax	Stage III vs Stage IV	4.00	0.437500	1.000000

Note: Pairwise comparisons are provided for transparency. No adjusted contrast should be interpreted independently of the limited block number and the corresponding omnibus test.

Supplementary Figures

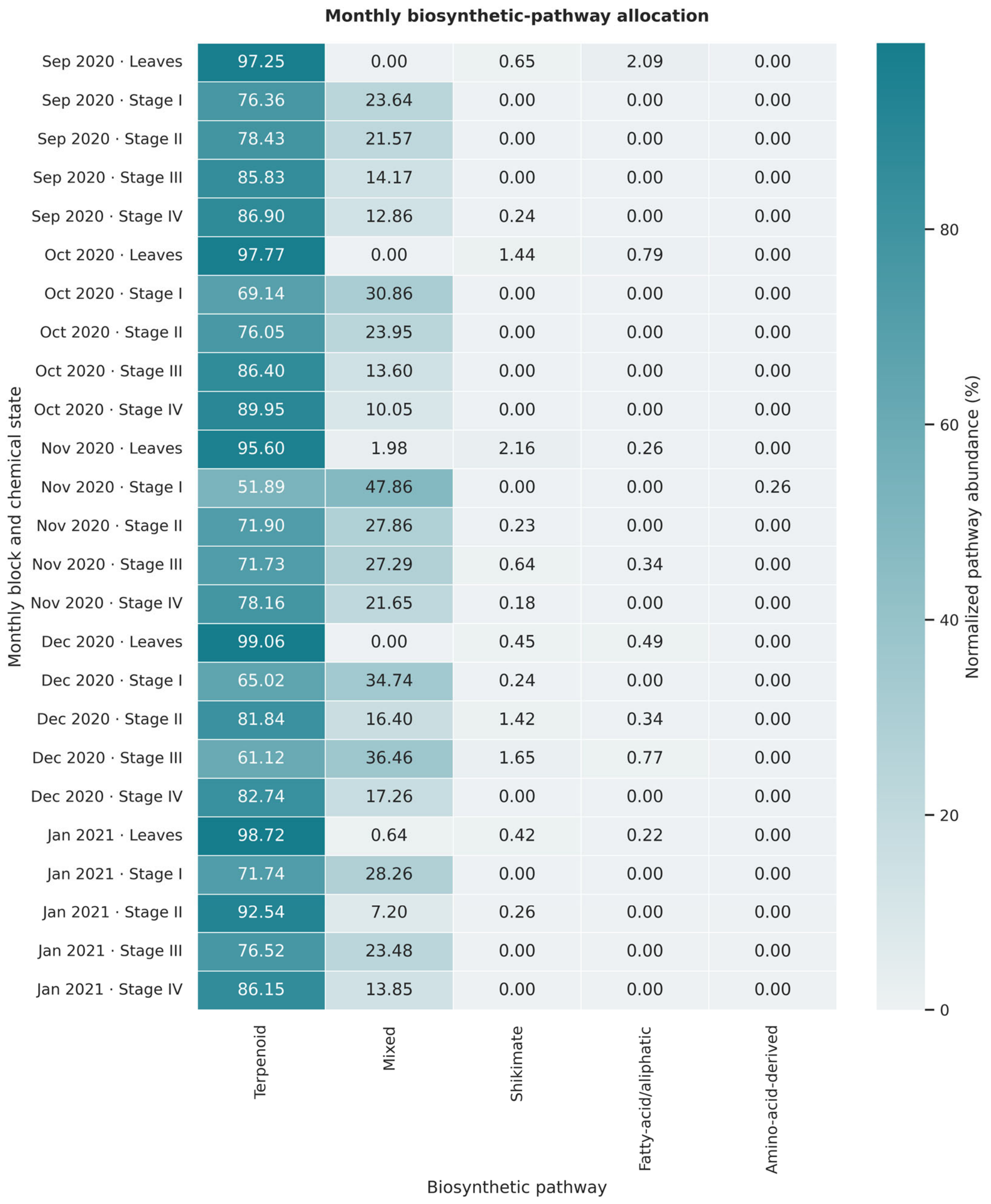

Figure S1. Monthly biosynthetic-pathway allocation across leaves and reproductive Stages I–IV. Values are normalized pathway percentages for each monthly block. Unlike the mean pathway allocation shown in the main article, this heatmap displays the complete block-specific structure used in the analyses.

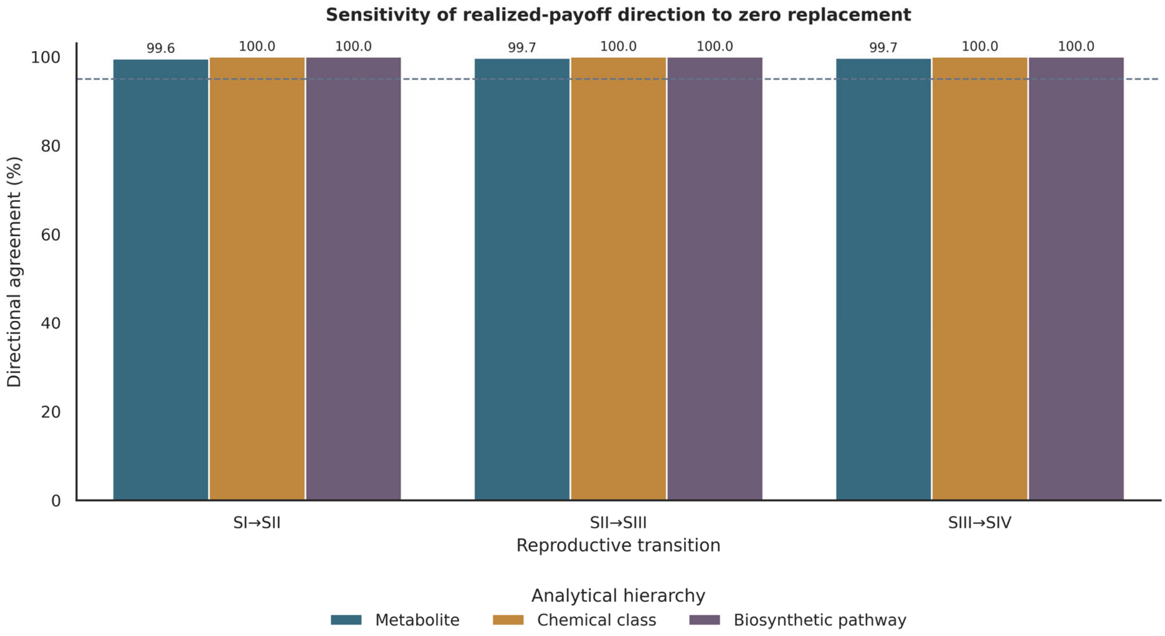

Figure S2. Sensitivity of realized-payoff direction to zero replacement at metabolite, chemical-class, and biosynthetic-pathway levels. Bars show the percentage of paired estimates retaining the same payoff sign when zeros were replaced by one-half versus one-tenth of the smallest positive abundance at the corresponding analytical hierarchy.

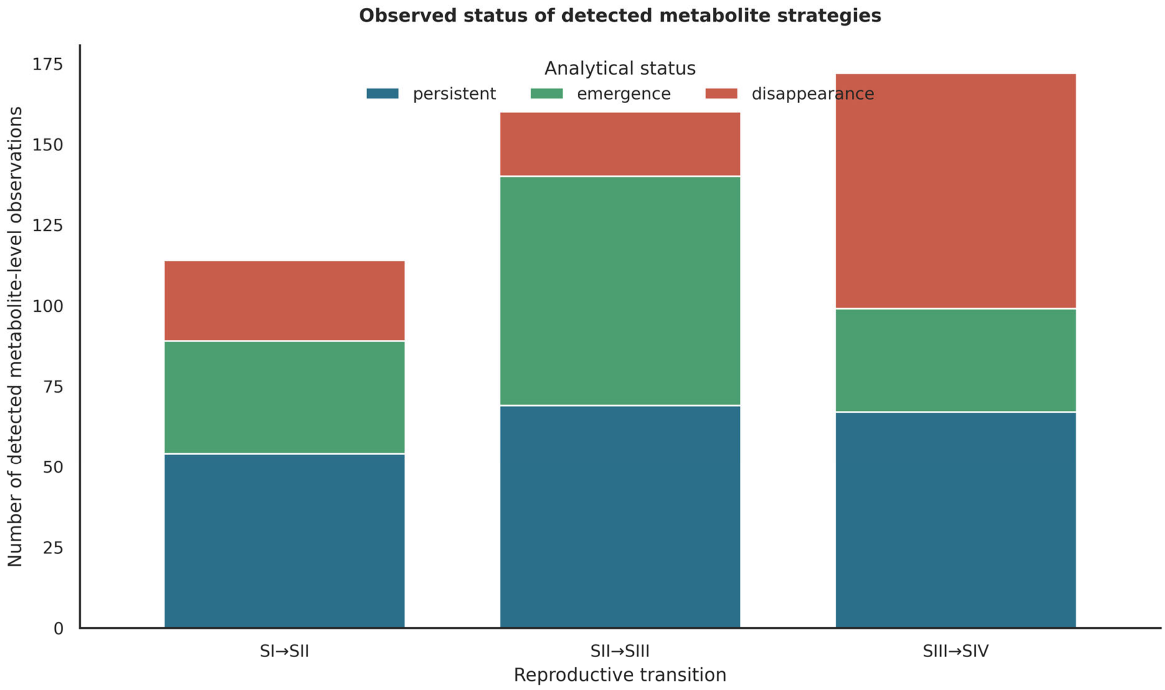

Figure S3. Observed analytical status of detected metabolite strategies across the five monthly blocks. Persistent strategies occurred in both adjacent states, emergent strategies were absent initially and detected subsequently, and disappearing strategies were detected initially and absent subsequently. Compound–transition combinations absent from both states were excluded from the figure.

References

[S1] de Brito Machado, D.; Felisberto, J.S.; Queiroz, G.A.d.; Guimarães, E.F.; Ramos, Y.J.; Moreira, D.d.L. From Leaves to Reproductive Organs: Chemodiversity and Chemophenetics of Essential Oils as Important Tools to Evaluate Piper mollicomum Kunth Chemical Ecology Relevance in the Neotropics. Plants 2024, 13, 2497. https://doi.org/10.3390/plants13172497.

[S2] Moreira, D.d.L.; Pereira, R.C.; Ramos, Y.J. Beyond Chemodiversity: A General Biosynthetic Diversity Index for Plant Metabolic Architecture in Ecology, Evolutionary, and Bioprospection. Molecules 2026, 31, 2188. https://doi.org/10.3390/molecules31122188.

[S3] Shannon, C.E. A Mathematical Theory of Communication. Bell Syst. Tech. J. 1948, 27, 379–423, 623–656.

[S4] Pielou, E.C. The Measurement of Diversity in Different Types of Biological Collections. J. Theor. Biol. 1966, 13, 131–144.

[S5] Taylor, P.D.; Jonker, L.B. Evolutionarily Stable Strategies and Game Dynamics. Math. Biosci. 1978, 40, 145–156. https://doi.org/10.1016/0025-5564(78)90077-9.

[S6] Friedman, M. The Use of Ranks to Avoid the Assumption of Normality Implicit in the Analysis of Variance. J. Am. Stat. Assoc. 1937, 32, 675–701.

[S7] Wilcoxon, F. Individual Comparisons by Ranking Methods. Biom. Bull. 1945, 1, 80–83.
